# Nonrandom domain organization of the *Arabidopsis* genome at the nuclear periphery

**DOI:** 10.1101/gr.215186.116

**Published:** 2017-07

**Authors:** Xiuli Bi, Ying-Juan Cheng, Bo Hu, Xiaoli Ma, Rui Wu, Jia-Wei Wang, Chang Liu

**Affiliations:** 1 Center for Plant Molecular Biology (ZMBP), University of Tübingen, Tübingen 72076, Germany;; 2 National Key Laboratory of Plant Molecular Genetics (NKLPMG), CAS Center for Excellence in Molecular Plant Sciences, Institute of Plant Physiology and Ecology (SIPPE), Shanghai Institutes for Biological Sciences (SIBS), Shanghai 200032, People's Republic of China;; 3 University of Chinese Academy of Sciences, Shanghai 200032, People's Republic of China;; 4 Department of Molecular Biology, Max Planck Institute for Developmental Biology, Tübingen 72076, Germany

## Abstract

The nuclear space is not a homogeneous biochemical environment. Many studies have demonstrated that the transcriptional activity of a gene is linked to its positioning within the nuclear space. Following the discovery of lamin-associated domains (LADs), which are transcriptionally repressed chromatin regions, the nonrandom positioning of chromatin at the nuclear periphery and its biological relevance have been studied extensively in animals. However, it remains unknown whether comparable chromatin organizations exist in plants. Here, using a strategy using restriction enzyme–mediated chromatin immunoprecipitation, we present genome-wide identification of nonrandom domain organization of chromatin at the peripheral zone of *Arabidopsis thaliana* nuclei. We show that in various tissues, 10%–20% of the regions on the chromosome arms are anchored at the nuclear periphery, and these regions largely overlap between different tissues. Unlike LADs in animals, the identified domains in plants are not gene-poor or A/T-rich. These domains are enriched with silenced protein-coding genes, transposable element genes, and heterochromatic marks, which collectively define a repressed environment. In addition, these domains strongly correlate with our genome-wide chromatin interaction data set (Hi-C) by largely explaining the patterns of chromatin compartments, revealed on Hi-C maps. Moreover, our results reveal a spatial compartment of different DNA methylation pathways that regulate silencing of transposable elements, where the CHH methylation of transposable elements located at the nuclear periphery and in the interior are preferentially mediated by CMT2 and DRM methyltransferases, respectively. Taken together, the results demonstrate functional partitioning of the *Arabidopsis* genome in the nuclear space.

The spatial organization of the genome within the nucleus is critical for many cellular processes (Van Bortle and Corces 2012). It is broadly accepted that the packing of chromatin inside the nucleus is not random, but is structured into several hierarchical levels (Gibcus and Dekker 2013). Cytological studies have shown that within the nucleus, each chromosome occupies a distinct domain known as the chromosome territory (CT). In *Arabidopsis thaliana*, CTs in interphase nuclei were unequivocally demonstrated with chromosome painting, which further revealed a predominantly random arrangement of CTs with respect to each other (Pecinka et al. 2004). Recent *Arabidopsis* Hi-C experiments also revealed many structural features of plant chromatin packing at both the chromosomal and the local levels (Feng et al. 2014b; Grob et al. 2014; Wang et al. 2015). At a gene level, several studies in *Arabidopsis* demonstrated an association between chromatin loops and gene transcriptional activity, which involves a diverse spectrum of regulatory factors (Crevillén et al. 2013; Liu et al. 2013; Ariel et al. 2014; Cao et al. 2014). On the other hand, the nonrandom location of chromatin segments with different biological properties within the nuclear space has long been documented. In *Arabidopsis*, densely packed and aggregated heterochromatin (chromocenters) is often tethered at the nuclear envelope, whereas telomeres often cluster and reside in the nuclear interior around the nucleolus (Armstrong et al. 2001; Fransz et al. 2002). Another recent study demonstrated structural and regulatory roles of chromatin associated with the nuclear matrix in plants (Pascuzzi et al. 2014). Together with experiments showing global rearrangement of chromatin triggered by various environmental and developmental factors, such as light (Barneche et al. 2014; Bourbousse et al. 2015), microbial infection (Pavet et al. 2006), and cell differentiation (Tessadori et al. 2007), all these studies highlight a close interaction between chromatin structure and function in plants.

Chromatin positioning at the nuclear periphery in animals has been extensively studied. The nuclear lamina is a layer of meshwork beneath the nuclear envelope, consisting of lamin and lamin-associated membrane proteins (Dechat et al. 2008). The nuclear lamin was found to participate in organizing chromatin structures by serving as an anchoring site for heterochromatin (for review, see Mattout et al. 2015). Genome-wide identification of chromatin regions associated with the nuclear lamina in animals led to the discovery of lamin-associated domains (LADs), which are large-sized, depleted of active histone marks, and low in gene density (Pickersgill et al. 2006; Guelen et al. 2008). On the other hand, the nuclear pore complex (NPC), a giant protein complex located at the nuclear envelope, has also been shown to play a role in tethering chromatin. Based on studies in yeast and several animal species, genes positioned close to the NPC tend to be highly transcribed (Strambio-De-Castillia et al. 2010).

Except for cytological studies showing a preferential association of chromocenters with the nuclear envelope, little is known about chromatin positioning at the nuclear periphery in plants. This is largely because plant genomes do not encode proteins with sequences similar to those of nuclear lamins in animals, although in several plant species, a meshwork similar to the nuclear lamina beneath the nuclear envelope has been observed (Ciska and Moreno Díaz de la Espina 2014). Nevertheless, over the past few years, a group of plant-specific nuclear matrix constituent proteins (NMCPs), such as CROWDED NUCLEI (CRWN) in *Arabidopsis*, have emerged as “plant lamina” components (Ciska and Moreno Díaz de la Espina 2014; Zhou et al. 2015). It appears that plant lamina components are distinct from those of animals, as another recently identified candidate, KAKU4, is also plant-specific (Goto et al. 2014). Moreover, NPC components have been systematically identified and investigated (Tamura et al. 2010; Tamura and Hara-Nishimura 2013; Parry 2015). These recent advances in knowledge provide opportunities for in-depth studies on various biological processes that occur at the plant nuclear periphery. In the present study, we identified and characterized *Arabidopsis* chromatin regions preferentially associated with the nuclear periphery on a genome-wide scale.

## Results

### RE-mediated ChIP reveals nonrandom chromatin distribution at the nuclear periphery

**Figure 1.**
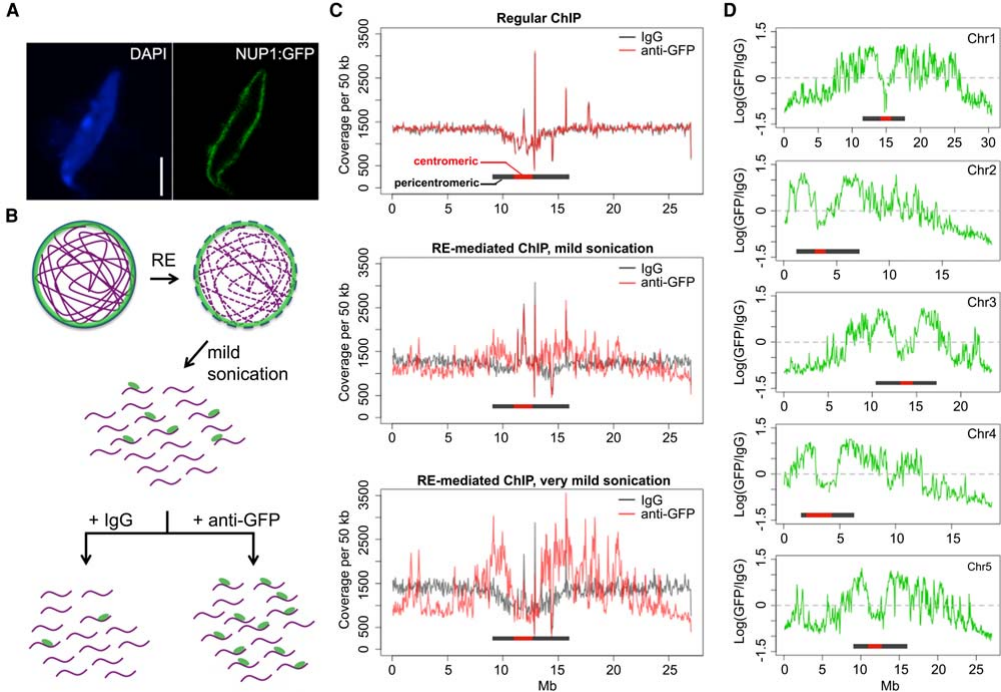
Identification of chromatin located at the nuclear periphery by RE-ChIP. (*A*) Localization of the NUP1:GFP protein in an *Arabidopsis* nucleus: (scale bar) 2 µm. (*B*) Procedures for RE-mediated ChIP with NUP1:GFP (green). Chromatin (purple lines) fragmentation and isolation are conducted with a combination of RE (restriction enzyme) digestion and mild sonication. (*C*) Normalized sequence coverage (50-kb window size) on Chromosome 5 from various ChIP experiments. The horizontal bars depict pericentromeric regions, within which centromeric regions are highlighted in red. (*D*) NUP1:GFP RE-mediated ChIP-seq signal (50-kb window size), represented as the log_2_ value of the ratio between normalized anti-GFP and IgG coverage, over all five chromosomes. Horizontal bars indicate the centromeric/pericentromeric regions, as in *C*.

As a part of the NPC basket, the *Arabidopsis* nucleoporin NUP1 (also known as NUP136) has been shown to specifically localize at the nuclear periphery (Lu et al. 2010; Tamura et al. 2010). In our first attempt, we sought to use the NUP1 protein, tagged with green fluorescent protein (GFP), to identify chromatin that directly interacts with NPC, which might be related to the “gene gating” events that have been demonstrated in yeast and animals (Blobel 1985; Strambio-De-Castillia et al. 2010). In agreement with previously reported results, the NUP1:GFP fusion protein was localized specifically at the nuclear envelope (Fig. 1A). With a regular chromatin immunoprecipitation (ChIP) method, however, we could not identify any chromatin regions enriched by NUP1:GFP, even with our ChIP-seq libraries being sequenced more deeply than typically needed for *Arabidopsis* (Supplemental Table S1). In contrast, a parallel ChIP experiment conducted on the same material, but with an antibody against RNA polymerase II, showed expected enrichment on a housekeeping gene (Supplemental Fig. S1), ruling out possible technical failures in our ChIP experiments. This negative result implied that NUP1:GFP did not directly interact with chromatin, or that such interactions, if they occurred, were not efficiently preserved by the crosslinking treatment in our ChIP experiment. To enhance the sensitivity of enriching chromatin loosely interacting with NUP1:GFP, we developed a restriction enzyme (RE)-mediated ChIP protocol, in which only mild sonication was applied to break the nuclei following digestion of chromatin with RE (Fig. 1B; Methods). In principle, compared to a regular ChIP method, in which chromatin is sheared into small fragments by much stronger sonic waves, RE-mediated ChIP causes less disruption to higher-order structures; in the case of NUP1:GFP ChIP, this method allows enrichment of the chromatin positioned around the NPC, or the nuclear periphery.

We performed two RE-mediated ChIP-seq trial experiments with different sonication intensities and examined the sequence coverage with a 50-kb window setting to gain an overview of the distribution of sequencing reads. Interestingly, the RE-mediated ChIP with NUP1:GFP (hereafter referred to as NUP1 RE-ChIP-seq) showed that chromatin in pericentromeric regions was generally enriched, whereas chromatin on the distal chromosome arms tended to be depleted (Fig. 1C,D). Moreover, we found many interstitial regions on the chromosome arms showing stronger contact with NUP1:GFP (e.g., an interval corresponding to 2.0–3.0 Mb on Chromosome 5) (Fig. 1D). In contrast, there were regions close to pericentromeric chromatin but that exhibited depleted contact with NUP1:GFP (e.g., an interval corresponding to 9.8–10.2 Mb on Chromosome 3) (Fig. 1D). These patterns, which were clearly correlated with sonication strength, could not be seen using our regular ChIP-seq assay (Fig. 1C, top). To validate the RE-ChIP method, we performed RE-ChIP-seq with anti-H3K9me2 (Supplemental Table S1) and compared the results with those derived from a regular ChIP-seq (Stroud et al. 2014). Consistent with the fact that the *Arabidopsis* pericentromeric heterochromatin is heavily marked by H3K9me2, our RE-ChIP clearly captured this epigenomic feature at a global level (Supplemental Fig. 2A). In addition, chromatin regions enriched by RE-ChIP-seq largely overlapped with those enriched by the regular ChIP-seq, by which >80% of chromatin regions enriched by ChIP-seq were found enriched in each RE-ChIP-seq replicate (Supplemental Fig. 2B,C), indicating the feasibility of the RE-ChIP method in capturing chromatin features in plants. Furthermore, selected regions showing higher NUP1 RE-ChIP signals could be confirmed with fluorescence in situ hybridization (FISH) (Supplemental Fig. 3). Taken together, our results suggest that certain chromatin regions on the *Arabidopsis* chromosome arms are preferentially found near the nuclear periphery.

It has been well demonstrated that chromocenters, which consist of the centromere and pericentromeric regions, are located preferentially at the nuclear periphery (Fransz et al. 2002; Fang and Spector 2005). It was interesting that chromatin from centromeres was not enriched in our NUP1 RE-ChIP-seq experiments (Fig. 1D). A possible scenario accounting for this observation is that NPCs (or at least NUP1-containing NPCs) are not evenly distributed at the nuclear envelope, such that the NPC density is lower in regions where chromocenters are anchored. For instance, kinetochore proteins interact with *Arabidopsis* centromeres in almost all stages of the cell cycle (Lermontova et al. 2013), and Gamma-tubulin Complex Protein 3-interacting proteins (GIPs) play essential roles in centromere assembly (Batzenschlager et al. 2015); these interactions might sequester centromeres away from NPCs. We examined nuclei in transgenic plants coexpressing NUP1:GFP and mCherry:CENH3, in which the latter was exclusively loaded to centromeres (Lermontova et al. 2006). We found that these two proteins displayed complementary patterns at the nuclear periphery, which explained our observation that centromeres were not enriched by NUP1:GFP (Supplemental Fig. 4A). Apart from this, consistent with the fact that pericentromeric chromatin is mostly found at the nuclear periphery, the chromatin regions belonging to the remaining pericentromeric regions showed the highest NUP1:GFP RE-ChIP signals (Supplemental Fig. 4B–F).

### Chromatin positioned at the nuclear periphery correlates with Hi-C map

**Figure 2.**
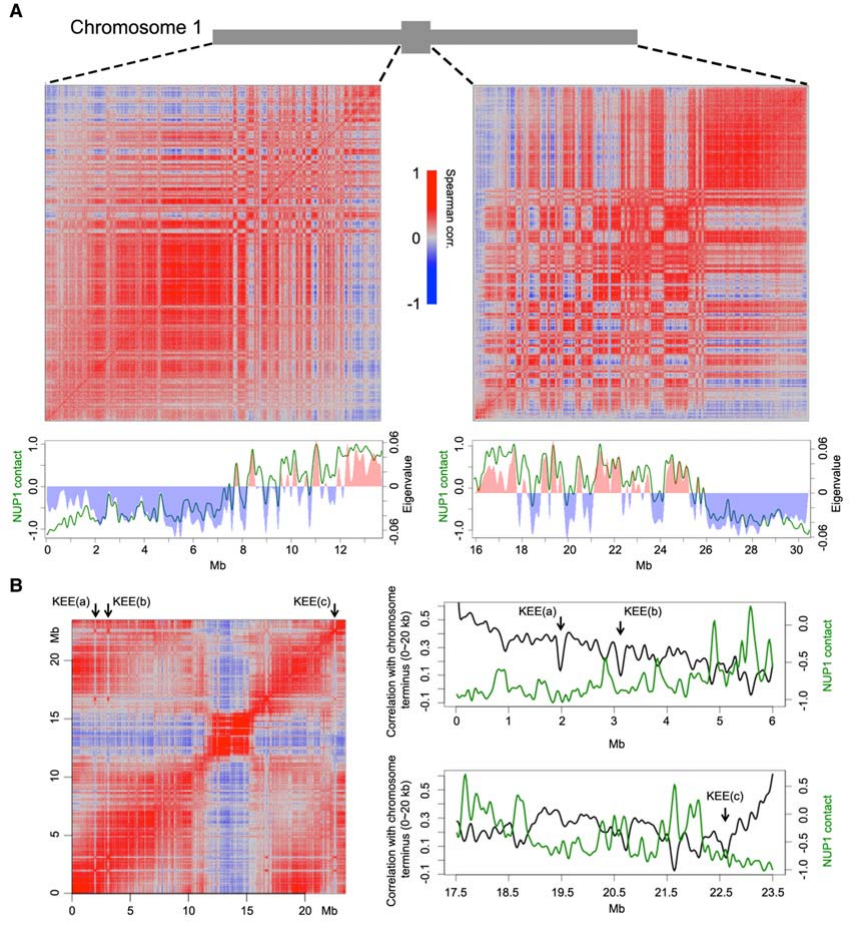
Correlation between chromatin anchored at the nuclear periphery and the Hi-C map. (*A*) Correlation between NUP1:GFP RE-ChIP-seq signal and Hi-C map. The Hi-C maps (normalized at 20-kb resolution) of the *left* and *right* Chromosome 1 arms are shown as Spearman correlation matrices, from which PCA was conducted; the eigenvalues of the first component are plotted *below* (red and blue bars) together with the NUP1:GFP signal (green lines, 20-kb window size), represented as the log_2_ value of the ratio between normalized anti-GFP and IgG coverage. (*B*) Anti-correlation between the telomeres and NUP1:GFP RE-ChIP-seq signal. The *left* panel shows a Spearman correlation matrix of Chromosome 3 derived from a Hi-C map at 20-kb resolution. Arrows depict KEE regions. The *right* panels highlight the 6-Mb distal chromosome regions, in which their correlation with the chromosome terminus (the first 20 kb of Chromosome 3) in the Hi-C map are shown as black curves. Green curves show the NUP1:GFP signal, as in *A*. Due to physical linkage, chromosome termini are expected to have strong colocalization with telomeres in the nucleus. In a Hi-C experiment, chromosome termini can be used to infer the spatial interactions between telomeres and other genomic regions.

The conformation of the *Arabidopsis* genome in the nuclear space has been recently revealed by several Hi-C experiments (Feng et al. 2014b; Grob et al. 2014; Wang et al. 2015). The Hi-C method combines chromatin conformation capture (3C) and high-throughput sequencing to generate a comprehensive view of how chromatin is folded (Lieberman-Aiden et al. 2009). Due to the nature of this method, the Hi-C data only contains information on the positioning of chromatin with respect to itself (chromatin folding). As our NUP1:GFP RE-ChIP-seq data focused on chromatin localization with respect to the nuclear boundary, we considered whether this nonoverlapping information could help us gain a better understanding of chromatin organization in the nuclei. Interestingly, we found that NUP1:GFP RE-ChIP-seq signals were strongly correlated with structural domains (SDs) derived from the *Arabidopsis* Hi-C map (Grob et al. 2014), which could be visualized when the chromosome arms were partitioned into two states using principal component analysis (PCA) (Fig. 2A). It is worth noting that such two-state classification, initially referred to as “AB” compartments, was found to be strongly correlated to the demarcation of active/repressed chromatin along the chromosome arms (Lieberman-Aiden et al. 2009; Grob et al. 2014), and a connection between animal LADs and the repressed compartment was recently shown on a global scale (Vieux-Rochas et al. 2015). We found that chromatin regions that had stronger contact with the nuclear periphery (with stronger NUP1:GFP RE-ChIP-seq signals) were mostly classified as the repressed compartment (Fig. 2A). Therefore, our results indicate that the “AB” compartments of *Arabidopsis* chromatin are associated with a radial axis of nuclei, further indicating that repressed chromatin is enriched at the nuclear periphery.

The *Arabidopsis* telomeres, except for those on the short arms of Chromosomes 2 and 4, are located around nucleoli (Armstrong et al. 2001; Fransz et al. 2002; Pontvianne et al. 2016). In general, chromatin on the distal chromosome arms exhibits a positive correlation with telomeres on a Hi-C map due to physical linkages, and this correlation gradually drops when the genomic distance increases. By checking the correlation between distal chromosome arms and telomeres, we observed local valleys, most of which colocalized with the local peaks of NUP1:GFP RE-ChIP-seq data (Fig. 2B). Because these local valleys of correlation indicate decreased chromatin interactions in the 3D space, this pattern agrees with the fact that *Arabidopsis* telomeres are preferentially found in the nuclear interior. We also found that some local valleys colocalized with interactive heterochromatic islands (IHIs)/knot-engaged elements (KEEs), which are regions showing strong intra- and inter-chromosomal interactions on Hi-C maps (Fig. 2B; Feng et al. 2014b; Grob et al. 2014). However, a comparison between KEEs and NUP1:GFP ChIP-seq did not reveal a connection between them (Supplemental Fig. 5), suggesting that the clustering of IHIs/KEEs does not preferentially occur at the nuclear periphery.

### Chromatin positioning at the nuclear periphery has similar patterns in different tissues

**Figure 3.**
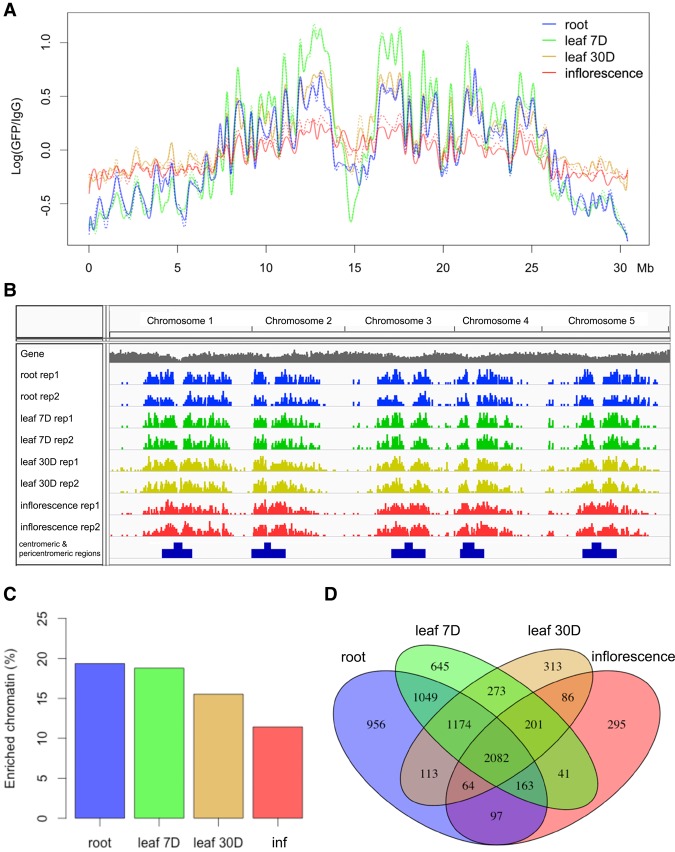
Genome-wide identification of NUP1-enriched regions in various tissues. (*A*) Signals of NUP1:GFP RE-ChIP-seq (20-kb window size), represented as the log_2_ value of the ratio between normalized anti-GFP and IgG sequence coverage over Chromosome 1. For each tissue, the solid and dotted lines depict two replicates. (*B*) Distribution of NUP1-enriched domains across the genome viewed with the Integrative Genomics Viewer browser (Robinson et al. 2011). (*C*) Percentage of NUP1-enriched genomic regions: (inf) inflorescence. (*D*) Venn diagram of genes enriched in four tissues.

Our finding of the nonrandom positioning of chromatin at the nuclear periphery prompted us to further investigate the extent to which these patterns vary among different plant tissues. In total, we examined NUP1:GFP RE-ChIP-seq data generated from four different tissues (Supplemental Table S1). The signal patterns, as well as enriched genes, between biological replicates were highly reproducible in each tissue (Fig. 3A; Supplemental Figs. 6–8). Interestingly, at a chromosomal level, NUP1:GFP RE-ChIP-seq data obtained from different tissues resembled each other (Fig. 3A; Supplemental Fig. 6). A common feature across these tissues was that chromatin close to the centromere was more frequently found at the nuclear periphery than was chromatin on the distal chromosome arms, which was reflected on a density plot showing the distribution of enriched chromatin segments (Fig. 3B). We noticed that in inflorescences, the difference in RE-ChIP-seq signal amplitudes between the pericentromeric regions and distal chromosome arms became much smaller, implying a lower selectivity in positioning specific chromatin regions at the nuclear boundary in reproductive tissues (Fig. 3A; Supplemental Fig. 6). Notably, for each plant tissue used in this study, the RE-ChIP output signal was the average of a mixture of different cell types. For the inflorescence tissue, compared to roots and leaves, the lower RE-ChIP signals around the pericentromeric regions might also be attributed to a dilution effect among different cells with divergent chromatin positioning patterns. Across different tissues, the regions enriched at the nuclear periphery covered 10%–20% of the genome (Fig. 3C), with median sizes of 7–12 kb (Supplemental Fig. 9; Supplemental Table S2). A clustering analysis showed that roots and leaves from 7-d-old seedlings formed a subgroup, although from a tissue-identity point of view, leaf tissues with different ages would be expected to be clustered together (Supplemental Fig. 7). Nevertheless, due to the similar RE-ChIP-seq signal profiles on a chromosomal scale (Fig. 3A; Supplemental Fig. 6), there were substantial overlaps of enriched chromatin regions between any two given tissues (Fig. 3B,D). These results suggest that although both the linear genome structure and the tissue identity contribute to the way chromatin is tethered at the nuclear periphery, the former is the primary determinant.

### Heterochromatic domains are enriched at the nuclear periphery in *Arabidopsis*

We next explored the genomic and epigenomic features associated with chromatin positioned at the nuclear periphery. As these chromatin regions were preferentially located around centromeres, we expected that features linked to the centromeric and pericentromeric regions would be enriched. To reduce such positional effects, we only included chromatin located at least 1 Mb from pericentromeric heterochromatin for all analyses described below (unless otherwise stated). Of note, our analyses in this study were not sensitive to a cutoff that we arbitrarily set. We obtained the same conclusions when we changed the cutoffs to 2 or 3 Mb, in which more genomic regions flanking the pericentromeric regions were excluded.

**Figure 4.**
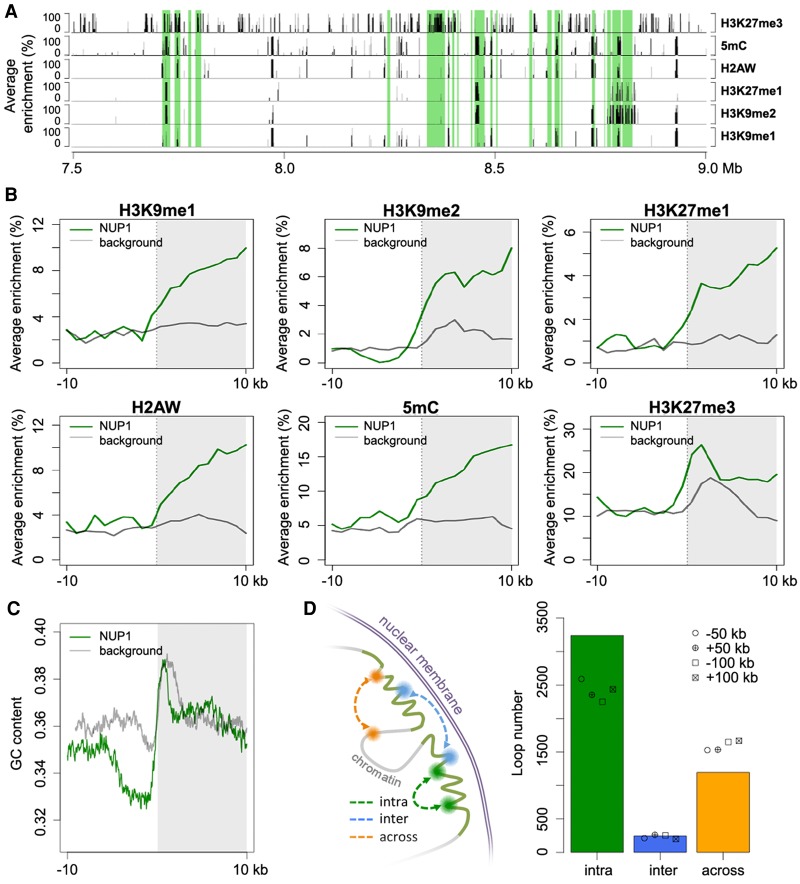
Epigenetic, genomic, and structural features of chromatin tethered at the nuclear periphery. (*A*) A representative genomic region from Chromosome 1 showing the distributions of NUP1-enriched chromatin identified from 7-d-old leaf tissues (shaded in green) and various epigenetic marks. Average enrichment means the percentage of regions (calculated from 100-bp windows) enriched for the respective epigenetic mark. (*B*,*C*) Epigenetic marks (*B*) and GC content (*C*) around NUP1-enriched domain borders, shown as a vertical line separating the white and gray blocks. For each plot, the area on the *right* indicates NUP1-enriched domains (although not all are larger than 10 kb). Average enrichment in *B* is defined as in *A*. The GC content in *C* is in a window size of 100 bp, with a step size of 20 bp. Because enrichment of gene bodies is found inward from NUP1-enriched domain boundaries (see Supplemental Fig. 12), for the background, we randomly picked 3000 genes with the same expression distribution profile as that of NUP1-enriched genes. For these control genes, we extracted the 20-kb regions flanking either their transcription start sites or their transcription termination sites, which were selected randomly. (*D*) Different types of chromatin loops associated with NUP1-enriched domains (including those in pericentromeric regions). Chromatin loops are from Liu et al. (2016). For both “intra” and “across,” the number of observed chromatin loops are significantly different (*P* < 2.2 × 10^−16^) relative to the permutation-based null distribution of the background, which was simulated by shifting the coordinates of NUP1-enriched domains ±50 kb or ±100 kb.

**Figure 5.**
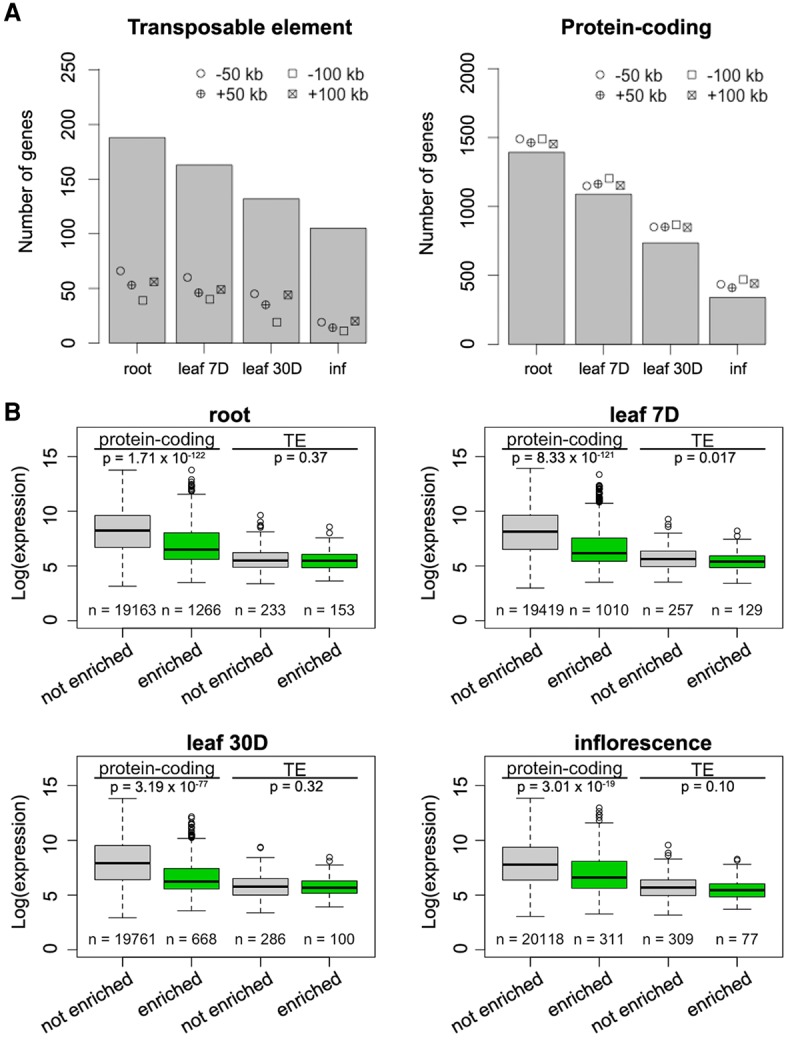
Enrichment of silenced genes at the nuclear periphery. (*A*) Number of TE genes (*left*) and protein-coding genes (*right*) enriched in different tissues. For each column, the observed number of genes is significantly different (*P* < 0.001) relative to the permutation-based null distribution of the background (generated as described in Fig. 4C): (inf) inflorescence. (*B*) Comparison of gene expression levels, which are from a normalized tilling array data set (Laubinger et al. 2008). The *P*-values indicate Mann-Whitney *U* test results.

Our association analyses of the epigenetic and genomic features around NUP1-enriched domain boundaries showed similar epigenetic landscapes compared to those of animal LADs, but there were significant differences in terms of other genomic features. For example, the NUP1-enriched domains were enriched with classic heterochromatic marks, such as H3K9 methylation, which has been linked to LADs (Towbin et al. 2012), and H3K27me3, which has been shown to enhance the association of chromatin to the inner nuclear membrane (Fig. 4A,B; Harr et al. 2015). Accordingly, the level of euchromatic marks was lower inside NUP1-enriched domains (Supplemental Fig. 10). On the other hand, in contrast to LADs, NUP1-enriched domains were neither substantially depleted with protein-coding genes (Fig. 5A; Supplemental Figs. 11, 12A) nor enriched with A/T-rich sequences (Fig. 4C). It is not clear whether the NUP1-enriched domain boundaries are bound with insulator proteins, as they have not yet been identified in plants. Nevertheless, we found that chromatin loops connecting regions inside and outside the NUP1-enriched domains were underrepresented; whereas chromatin interactions restricted within one NUP1-enriched domain were overrepresented (Fig. 4D). To a certain extent, this pattern is analogous to that of topologically associating domains (TADs), which are predominant structural units of higher-order chromatin architecture in many metazoan genomes (Dixon et al. 2012). Typically, chromatin interactions within a TAD are observed more often than expected, whereas those across TAD boundaries are underrepresented. As chromatin inside NUP-enriched domains showed suppressed interactions with outside regions in our study, from a spatial point of view, these domains represented structures that were isolated from their surroundings.

### The *Arabidopsis* nuclear periphery is enriched for repressed chromatin

Based on gene annotations, we found that in all tissues, transposable element (TE) genes and pseudogenes were enriched compared to the control sets, which were simulated by shifting the coordinates of the enriched regions a certain distance upstream or downstream (Fig. 5A; Supplemental Fig. 11; Supplemental Table S3). We considered these control sets to be more appropriate than random permutations, as they maintained the distribution pattern of the enriched regions across the genome. In terms of transcriptional activity, the enriched protein-coding genes clearly had lower expression levels (Fig. 5B), which aligned with our finding that chromatin positioned at the nuclear periphery was generally repressed (Figs. 2, 4). Interestingly, for genes with a transcription direction toward the interior of the NUP1-enriched domains, we observed a higher occurrence of transcription start sites (TSSs) of genes with low transcription levels around domain boundaries (Supplemental Fig. 12B), suggesting a role of gene bodies in demarcating these chromatin domains. On the other hand, TE genes enriched at the nuclear periphery did not show lower expression levels than those that were not enriched (Fig. 5B). Instead, these two types of TE genes differed in terms of their lengths and locations with respect to protein-coding genes; TE genes located at the nuclear periphery were significantly longer and were located further from protein-coding genes (Supplemental Fig. 13). Additionally, TE genes enriched at the nuclear periphery showed a higher average level of heterochromatin marks, such as H3K9me2, H3K27me1, and DNA methylation (Supplemental Fig. 14). In terms of classes, two class-II TE genes (MuDR and CACTA-like) were more likely to be found at the nuclear periphery (Supplemental Fig. 15). Taken together, the *Arabidopsis* nuclear periphery defines a domain of transcriptional repression enriched for TE genes and transcriptionally inactive protein-coding genes.

### Positioning of TEs at the nuclear periphery correlates with different silencing pathways

**Figure 6.**
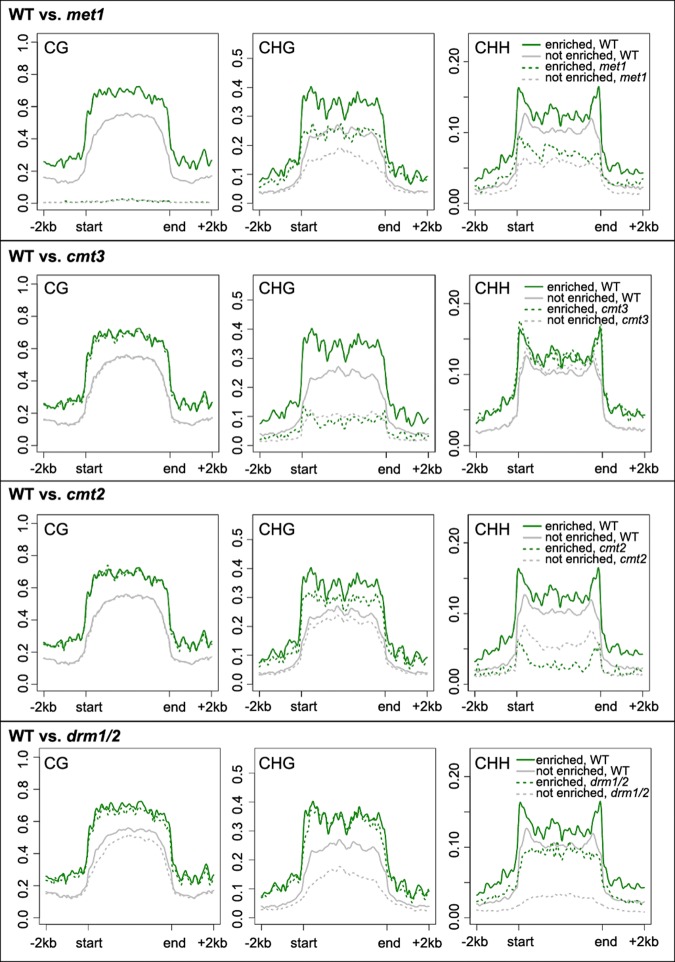
Comparison of DNA methylation over TEs. Patterns of TE DNA methylation (CpG, CHG, CHH) in wild-type (WT) and mutants. The grouping of TEs is according to the enrichment results of NUP1:GFP RE-ChIP-seq from 30-d-old leaf tissues. The methylation ratio is calculated in 100-bp windows. The signal over each TE is linearly transformed so that the boundaries of all TEs are aligned.

Having shown that the nuclear peripheral zone was repressed, we next investigated whether it was connected to silencing of TEs. DNA methylation in the CG, CHG, and CHH (H representing any nucleotide except G) sequence context plays a crucial role in regulating expression and transposition of TEs. We noticed that TEs enriched at the nuclear periphery had a higher DNA methylation level in all sequence contexts (Fig. 6). We next examined several DNA methylation mutants by asking how the corresponding types of methylation would change in these two types of TEs. Regardless of TE location in the nuclear space, mutations impairing CG or CHG methylation showed similar effects (Fig. 6). Interestingly, when comparing the CHH methylation patterns, we found that TEs located at the nuclear periphery lost more DNA methylation in the *cmt2* mutant; in contrast, TEs not located at the nuclear periphery lost more DNA methylation in the *drm1*/2 double mutant (Fig. 6). These patterns were also observed when we focused on TEs located in the pericentromeric regions (Supplemental Fig. 16). CHH methylation over TE bodies is mediated by two partially overlapping pathways: RNA-directed DNA methylation (RdDM) and RdDM-independent (Zemach et al. 2013; Stroud et al. 2014). However, it is not clear how these two pathways branch to target different TEs (for review, see Sigman and Slotkin 2016). DOMAINS REARRANGED METHYLASE 1 (DRM1) and DRM2 are responsible for CHH methylation in the RdDM pathway, whereas CHROMOMETHYLASE 2 (CMT2) is required for the RdDM-independent pathway (Cao et al. 2003; Stroud et al. 2013; Zemach et al. 2013). Our results reveal a spatial association between TE locations and the demand on different CHH methylation pathways, in which CHH methylation of TEs located at the nuclear periphery tends to be more dependent on CMT2, whereas the other type of TE relies more on RdDM.

## Discussion

It has long been recognized that the *Arabidopsis* chromocenters are preferentially found at the nuclear periphery, but it was unclear whether such nonrandom localization was restricted to chromocenters. The present study demonstrates that the peripheral zone of the *Arabidopsis* nucleus is also enriched with interstitial regions on the chromosome arms, which are mainly heterochromatic. This is reflected by the fact that these regions have higher percentages of TE genes and silenced protein-coding genes (Figs. 4, 5). In this regard, the nuclear periphery in plants is a functional compartment for docking repressed chromatin; therefore, the biological properties of the nuclear periphery in eukaryotes are highly conserved. An intriguing question is which factors are involved in specifically tethering chromatin to the plant nuclear periphery. In animals, lamins and lamin-associated proteins have been identified as key factors involved in these events (for review, see Harr et al. 2016). Unfortunately, to identify the counterparts in plants, a strategy based on a protein-sequence similarity search might be of little use compared to forward genetics approaches, because plants lack orthologs of lamins and most lamin-associated proteins (Ciska and Moreno Díaz de la Espina 2014). Nevertheless, the CRWN and KAKU4 proteins in *Arabidopsis* have been suggested as plant lamin candidates, and *CRWN* mutations result in altered chromocenter structure (Wang et al. 2013). In the *crwn4* mutant, although the chromocenters decondensed, the regions corresponding to the 5S RNA repeats remained anchored at the nuclear periphery (Wang et al. 2013). This suggests the need to investigate higher-order *crwn* mutants to clarify their potential roles in tethering chromatin.

In this study, many highly expressed genes were also found to be enriched at the nuclear periphery (Supplemental Table S3), which could be at least attributed to following reasons. First, both the gene expression and RE-ChIP-seq experiments were conducted on tissues with a certain degree of cell-type heterogeneity; therefore, even if active transcription and positioning at the nuclear periphery are mutually exclusive, both events might be eventually captured at a gene locus in a mixed cell population. Second, the nuclear periphery does not absolutely inhibit transcription. This has been demonstrated in human cells by tracking the expression of a pool of genes after artificially anchoring them to the nuclear envelope; only a subset of the targeted genes showed reduced expression (Finlan et al. 2008). Specifically in *Arabidopsis*, a recently reported case study on the *CHLOROPHYLL A/B BINDING* (*CAB*) *PROTEIN* locus showed that it is repositioned from the nuclear interior to the nuclear periphery together with robust transcriptional activation in response to light stimuli (Feng et al. 2014a). Third, in plants, there may be “gene gating” events that position actively transcribed genes at the nuclear periphery through interactions with nucleoporins (Blobel 1985). Several potential interactions between transcription regulators (such as the TREX2 complex and SUMO proteases) and NPC have recently been discussed (Parry 2015). Although we did not detect any direct binding of NUP1 to chromatin with a regular ChIP-seq method, it remains unknown whether other NPC components directly interact with chromatin.

Interestingly, the RE-ChIP signals from the root tissue, which consisted of nonmesophyll cells, were highly similar to those from leaves, with the majority cell type being mesophyll cells (Fig. 3A,B). Compared to other tissues, RE-ChIP signals from inflorescences showed a much lower extent of enrichment for chromatin at the nuclear periphery, which might be attributed to a dilution effect due to cell-type heterogeneity. Overall, the chromatin regions positioned at the nuclear periphery in plants tend to be conserved among different tissues, suggesting that in general, the linear genome per se contributes substantially to how it is deployed with respect to the nuclear peripheral zone on the chromosomal scale. By showing a tight association between this pattern and the “AB” compartment derived from Hi-C maps (Fig. 2A), we provide an additional way to visualize and understand plant Hi-C maps in the context of the nuclear space. Although Hi-C maps from *Arabidopsis* roots or inflorescences are not presently available, we suspect that the chromatin packing in these tissues would follow a scheme in common with that of leaves, and all these Hi-C maps would be strongly correlated with each other on a global level.

Having “AB” compartments in the nuclear space implies the existence of a radial gradient, offering a spatial specificity with which different regulatory pathways can regulate chromatin activity. Globally, this is reflected by the observation that chromocenters and telomeres preferentially reside at the nuclear periphery and nuclear interior (around the nucleolus), respectively (Fransz et al. 2002). By showing that TEs are selectively tethered at the nuclear periphery, our results reveal additional features of this spatial compartment. The differential loss of CHH DNA methylation on TEs in the RdDM and RdDM-independent mutants implies a spatial preference of these two TE-silencing pathways, in which RdDM is under more demand in the nuclear interior (Fig. 6). This correlates with observations that many small RNA pathway components are concentrated around nucleoli in *Arabidopsis* (Li et al. 2008; Pontes et al. 2013). From a spatial point of view, our results provide insights into how these two silencing pathways might collaborate to regulate TEs (Zemach et al. 2013), as well as how certain components of one pathway interact with each other (e.g., a recently reported positive feedback loop between Pol IV-dependent small RNA biogenesis and DRM2-dependent CHH methylation (Li et al. 2015)). It would also be of great interest to further investigate the possible dynamic locations of TEs in mutants of TE-silencing pathways.

Conventionally, chromatin regions that are preferentially tethered to the nuclear periphery could be identified by the ChIP method, such as those showcased in recent studies on animal nuclear lamin A and lamin B (Kubben et al. 2012; Lund et al. 2013; Sadaie et al. 2013; Shah et al. 2013). Additionally, an alternative approach is to anchor a modification enzyme on the nuclear envelope and trace its footprint on the genome (Kind et al. 2013). This method, which utilizes a DNA adenine methyltransferase (Dam) that methylates DNA on the *N*^6^-adenine residue, has been applied to *Arabidopsis* to identify targets of LIKE HETEROCHROMATIN PROTEIN 1 (LHP1) as a complementary approach to the conventional ChIP method (Zhang et al. 2007). A potential limitation of these approaches is that the protein of interest must be in close contact with chromatin. In plants, however, these proteins’ identities remain unknown. By performing RE-mediated chromatin fragmentation in combination with mild sonication, our RE-ChIP protocol alleviates the requirement that proteins of interest must directly interact with chromatin, because in principle the RE-ChIP would better protect higher-order structures from destruction by strong sonic waves and would permit recovery of chromatin in the proximity of a protein of interest even when the interaction is not direct. In our opinion, this is a feasible method for identifying chromatin, if it is positioned close to other subnuclear structures, such as the nuclear matrix, nucleolus, and various nuclear bodies. The RE-ChIP method uses a restriction enzyme to digest chromatin; therefore, the chromatin fragmentation pattern is not random and is dependent on both the restriction-cutting site density and the digestion efficiency (Wang et al. 2015). Compared to regular ChIP methods, RE-ChIP cannot achieve resolution at the nucleosomal level and is not suitable for genome-wide identification of narrow peaks, such as the typical binding sites of transcription factors.

## Methods

### Plant material

*Arabidopsis thaliana* transgenic plants *NUP1:GFP* in the *nup136-1* (Salk_104728) background were grown at 23°C in long days (16 h light/8 h dark) on half-strength Murashige and Skoog (MS) medium supplemented with 1% sucrose and 0.3% Phytagel. The aerial and root tissues of 7-d-old seedlings were harvested at Zeitgeber time (ZT) 6 h. Other tissues, including 30-d-old leaf and inflorescence with flower bud up to stage 9 (Smyth et al. 1990), were collected from plants grown in growth rooms under long days at 23°C.

### Plasmid construction

*NUP1:GFP* was constructed with an overlapping PCR strategy. The genomic fragment spanning 600 bp upstream of *NUP1* to the *NUP1* stop codon was amplified with primers 5′-GTTCGTTAGACTGGTTTAGGT-3′ and 5′-TTTCTTCCTGGTGGATTTCTT-3′; the genomic fragment spanning the *NUP1* stop codon to 150 bp downstream from *NUP1* was amplified with primers 5′-TTTGGAGAAGAAGGCTTCTCT-3′ and 5′-TAAGAAAAACACATTGTTCAAG-3′; and GFP cDNA was amplified with primers 5′-AAGAAATCCACCAGGAAGAAAGCGGCCGCTGTGAGCAAGGG-3′ and 5′-CTTGAACAATGTGTTTTTCTTAAGATCCACCAGTATCCTCAC-3′. These PCR products were mixed and assembled by overlapping PCR and amplified with primers 5′-GTTCGTTAGACTGGTTTAGGT-3′ and 5′-TTTGGAGAAGAAGGCTTCTCT-3′. The final PCR product, in which *GFP* was fused with *NUP1*, was cloned into a Gateway- compatible pGREEN-IIS binary destination vector (Karlsson et al. 2015). Similarly, to make the mCherry:CENH3 fusion protein, mCherry was amplified with primers 5′-GTAAAAATCAATGGCCATCATCAAGGAGTT-3′ and 5′-ACGCGATGCTTGGTTCTCGCACCGCCACCCTTGTACAGCTCGTCCATGC-3′, cenH3 (AT1G01370) was amplified with primers 5′-GCGAGAACCAAGCATCGCGT-3′ and 5′-TCACCATGGTCTGCCTTTTC-3′, these PCR products were assembled by overlapping PCR and amplified with primers 5′-GTAAAAATCAATGGCCATCATCAAGGAGTT-3′ and 5′-TCACCATGGTCTGCCTTTTC-3′. The PCR product was cloned into a Gateway-compatible pGREEN-IIS binary destination vector containing a 35S promoter (Karlsson et al. 2015).

### RE-ChIP-seq library preparation

Tissues were collected and fixed under vacuum for 30 min with 1% formaldehyde in MC buffer (10 mM potassium phosphate, pH 7.0; 50 mM NaCl; 0.1 M sucrose) at room temperature. After fixation, tissues were incubated at room temperature for 5 min under vacuum in MC buffer with 0.15 M glycine. Nuclei from 0.5 g fixed material were used for each round of ChIP. Nuclei were isolated as described (Wang et al. 2015). Nuclei were permeabilized through incubation with 150 µL 0.5% SDS for 5 min at 62°C, and SDS was quenched with addition of 75 µL of 10% Triton X-100. Following the nuclei permeabilization treatment, chromatin was digested overnight with 150 units DpnII at 37°C, which was deactivated the next morning for 20 min at 62°C. Next, nuclei were collected after spinning at 1000*g* for 3 min, and suspended with 1 mL sonication buffer (10 mM potassium phosphate, pH 7.0; 0.1 mM NaCl; 0.5% Sarkosyl; 10 mM EDTA) and sheared by sonication with a Covaris S220 instrument (set at 20dc, 1i, 200cpb, 15 sec). The sonicated sample was centrifuged at 14,000 rpm for 5 min, and the supernatant was mixed with 100 µL 10% Triton X-100. Next, the sheared chromatin was mixed with an equal volume of IP buffer (50 mM Hepes, pH 7.5; 150 mM NaCl; 5 mM MgCl_2_; 10 µM ZnSO_4_; 1% Triton X-100; 0.05% SDS) and then equally divided and incubated with anti-GFP antibody (Abcam, ab290) or normal rabbit IgG (Santa Cruz, sc-2027), respectively. After overnight incubation at 4°C, 10 µL Protein A/G magnetic beads (Pierce) were added and incubated for 2 h at 4°C. The beads were washed at 4°C as follows: 3× with IP buffer, 1× with IP buffer having 500 mM NaCl, and 1× with LiCl buffer (0.25 M LiCl; 1% NP-40; 1% deoxycholate; 1 mM EDTA; 10 mM Tris pH 8.0), for 5 min each. Chromatin retained on beads was incubated in 200 µL elution buffer (50 mM Tris, pH 8.0; 200 mM NaCl; 1% SDS; 10 mM EDTA) for 6 h at 65°C, followed by Proteinase K treatment for 1 h at 37°C. DNA was extracted with a standard phenol-chloroform method. To increase sequence diversity at the ends of DNA, the immunoprecipitated DNA was incubated with dsDNA Fragmentase (NEB) for 25 min at 37°C, which randomly cut DNA into ∼100- to 200-bp fragments. The digested DNA was purified with AMPure XP beads (Beckman Coulter), and all subsequent end repairing, A-tailing, adaptor ligation, library amplification steps were done through following a standard protocol (Illumina). The final libraries were sequenced on an Illumina HiSeq 3000 instrument with 2×150-bp reads.

### Sequencing reads analysis

Paired-end reads were aligned against the *Arabidopsis thaliana* reference genome (TAIR10) using Bowtie 2 v2.2.4 (Langmead and Salzberg 2012) with a “very sensitive” mapping mode. For each replicate, the mapped reads were analyzed by SICER v1.1 (Zang et al. 2009) to call enriched regions (parameters: W = 1000; G = 3000; FDR < 0.01). For each type of tissue, regions shared between the two replicates were extracted, which were classified as domains enriched at the nuclear periphery (or NUP1-enriched domains). The *Arabidopsis* gene annotation was retrieved from Ensembl Genomes (ftp://ftp.ensemblgenomes.org/) (release-24) (Kersey et al. 2016). A gene was claimed enriched if >80% of its transcribed region overlapped with NUP1-enriched domains.

### FISH

The FISH experiment was performed as previously published (Prieto et al. 2007; Wegel et al. 2009) with modifications, in which instead of biotin-16-dUTP, dinitrophenol-11-dUTP (DNP-11-dUTP) (PerkinElmer) was used to label BAC probes in the nick translation reaction. Slide pretreatment, hybridization, and post-hybridization wash were carried out as described (Prieto et al. 2007). Detection of the digoxigenin-11-dUTP was done with 1:1000 mouse anti-digoxin antibody (Sigma, D-8156) and followed by 1:150 goat anti-mouse antibody coupled to Alexa Fluor 488 (Invitrogen, A11017). Detection of the DNP-11-dUTP was done with 1:500 rabbit anti-dinitrophenyl antibody (Invitrogen, A6430) and followed by 1:150 goat anti-rabbit antibody coupled to Alexa Fluor 546 (Invitrogen, A-11071). After the final wash step, slides were mounted with SlowFade Diamond Antifade Mountant with DAPI (Thermo Fisher Scientific).

### Fluorescence microscopy

Confocal images were acquired with the Leica SP8 AOBS system. The detection of various fluorophores (DAPI, Alexa Fluor 488, and Alexa Fluor 546) and fluorescent proteins (GFP and mCherry) was according to the default settings. Image processing was done with the Fiji software and final assembly in Photoshop. For the distance measurement of FISH signals, first, a *Z*-stack image was obtained by maximum projection of signals from five optical sections. Then, the nuclear edge was defined by adjusting the threshold of DAPI channel, and the distance between a FISH signal and the nuclear edge was determined as described (Feng et al. 2014a). Only nuclei containing hybridization signals of both probes were included in analyses.

### Published genomic data

Data for gene expression in various tissues were from Laubinger et al. (2008), bisulfite sequencing from Stroud et al. (2013), Hi-C matrix (20-kb window setting) from Wang et al. (2015), and chromatin loops and other processed epigenetic data sets from Liu et al. (2016). Definition of centromeric regions (Chr 1, ∼14.08–15.61 Mb; Chr 2, ∼2.93–3.95 Mb; Chr 3, ∼13.16–14.55 Mb; Chr 4, ∼2.00–4.26 Mb; and Chr 5, ∼10.93–12.66 Mb) was according to coordinates of BAC clones on the TAIR10 genome that overlapped with the boundaries of estimated centromeric regions (The Arabidopsis Genome Initiative 2000). The definition of pericentromeric heterochromatin (Chr 1, ∼11.5–17.7 Mb; Chr 2, ∼1.1–7.2 Mb; Chr 3, ∼10.3–17.3 Mb; Chr 4, ∼1.5–6.3 Mb; and Chr 5, ∼9.0–16.0 Mb) was according to Stroud et al. (2013).

## Data access

All sequence data from this study have been submitted to the NCBI Sequence Read Archive (SRA; http://www.ncbi.nlm.nih.gov/sra) under accession number SRP079108.

## Supplementary Material

Supplemental Material
