# Supplementary material for: Nonrandom domain organization of the Arabidopsis genome at the nuclear periphery
Source: Genome Res. 2017 Jul;27(7):1162–73. doi: 10.1101/gr.215186.116 (PMC5495068; doi:10.1101/gr.215186.116)
Supplement: Supplemental Material [file supp_27_7_1162__index.html]

Nonrandom domain organization of the Arabidopsis genome at the nuclear periphery — Supplemental Material 

# Nonrandom domain organization of the *Arabidopsis* genome at the nuclear periphery

## Supplemental Material

- Supplemental\_figures\_and\_legends.pdf
- Supplemental\_Table\_S1.docx
- Supplemental\_Table\_S2.xlsx
- Supplemental\_Table\_S3.txt
- Supplemental\_Table\_S4.docx
