# Supplementary material for: Nonrandom domain organization of the Arabidopsis genome at the nuclear periphery
Source: Genome Res. 2017 Jul;27(7):1162–73. doi: 10.1101/gr.215186.116 (PMC5495068; doi:10.1101/gr.215186.116)
Supplement: Supplemental Material [file supp_gr.215186.116_Supplemental_figures_and_legends.pdf]

Non-random Domain Organization of the *Arabidopsis* Genome at the Nuclear Periphery

Xiuli Bi, Yingjuan Cheng, Bo Hu, Xiaoli Ma, Rui Wu, Jia-Wei Wang, and Chang Liu

Supplemental references

Feng S, Cokus SJ, Schubert V, Zhai J, Pellegrini M, Jacobsen SE. 2014. Genome-wide Hi-C analyses in wild-type and mutants reveal high-resolution chromatin interactions in Arabidopsis. *Mol Cell* **55:** 694-707.

Grob S, Schmid MW, Grossniklaus U. 2014. Hi-C analysis in Arabidopsis identifies the KNOT, a structure with similarities to the flamenco locus of Drosophila. *Mol Cell* **55:** 678-693.

Liu C, Teo ZW, Bi Y, Song S, Xi W, Yang X, Yin Z, Yu H. 2013. A conserved genetic pathway determines inflorescence architecture in Arabidopsis and rice. *Dev Cell* **24:** 612-622.

Liu C, Wang C, Wang G, Becker C, Zaidem M, Weigel D. 2016. Genome-wide analysis of chromatin packing in Arabidopsis thaliana at single-gene resolution. *Genome Res* **26:** 1057-1068.

Robinson JT, Thorvaldsdottir H, Winckler W, Guttman M, Lander ES, Getz G, Mesirov JP. 2011. Integrative genomics viewer. *Nat Biotechnol* **29:** 24-26.

Stroud H, Do T, Du J, Zhong X, Feng S, Johnson L, Patel DJ, Jacobsen SE. 2014. Non-CG methylation patterns shape the epigenetic landscape in Arabidopsis. *Nat Struct Mol Biol* **21:** 64-72.

Wang C, Liu C, Roqueiro D, Grimm D, Schwab R, Becker C, Lanz C, Weigel D. 2015. Genome-wide analysis of local chromatin packing in Arabidopsis thaliana. *Genome Res* **25:** 246-256.

Zang C, Schones DE, Zeng C, Cui K, Zhao K, Peng W. 2009. A clustering approach for identification of enriched domains from histone modification ChIP-Seq data. *Bioinformatics* **25:** 1952-1958.

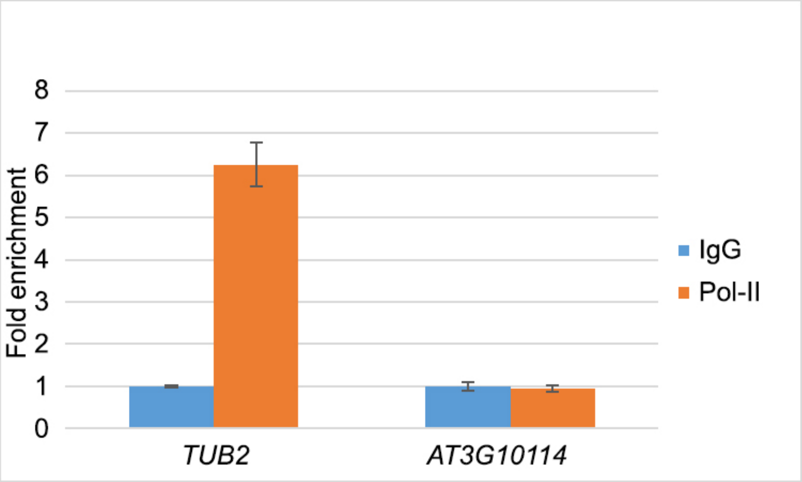

Supplemental Figure S1. Control ChIP experiment with antibodies against RNA-polymerase II. Chromatin fragments isolated from *NUP1:GFP nup136-1* plants according to the regular ChIP protocol (as in the upper panel in Fig. 1C) were immunoprecipitated with anti-Pol II antibody (Abcam ab5408). A genomic fragment of housekeeping gene *TUBULIN2* (*TUB2*) transcribed region and an intergenic region 2.2 kb away from *AT3G10114* were used as positive and negative controls, respectively. The relative abundance of these two genomic fragments was determined via normalization with respect to a third genomic region, an intergenic region 100 bp away from *AT2G24890*, which was free of Pol-II binding according to our published ChIP-seq data (Liu et al. 2016). Calculation of fold enrichment with quantitative real-time PCR was performed as described previously (Liu et al. 2013). Error bars mean standard deviation. All primers sequences used for ChIP-qPCR are listed in Supplemental Table S4.

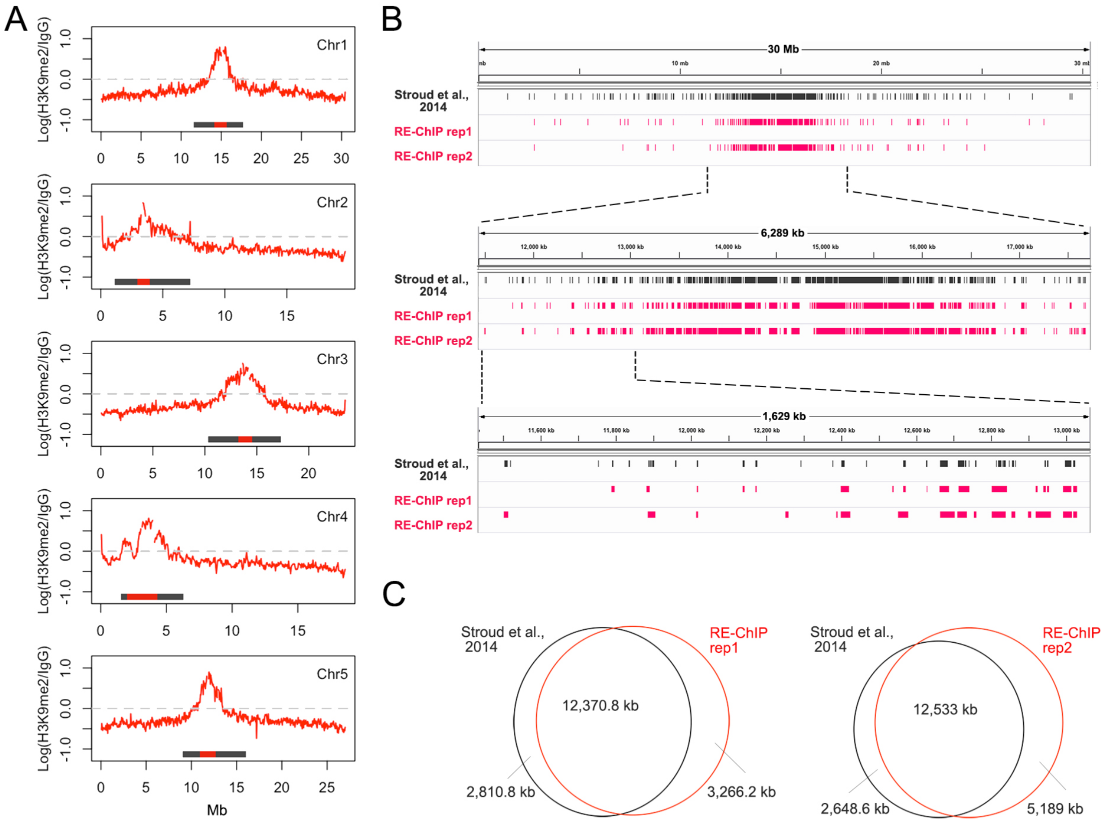

Supplemental Figure S2. Comparison between RE-ChIP and regular ChIP.
(A) RE-ChIP-seq signals with anti-H3K9me2 antibodies (Abcam ab1220). Leaves of 7-day-old wild-type seedlings were used. Labels are the same as in Figure 1D. (B) Distribution of enriched chromatin regions on chromosome 1, viewed with the Integrative Genomics Viewer browser (Robinson et al. 2011). For the regular ChIP-seq data (Stroud et al. 2014), the enriched regions were identified with the SICER v1.1 program with window size and gap size set as 200 and 400, respectively (Zang et al. 2009). For the RE-ChIP, the enriched regions were identified as described in Methods. The middle and lower panels show zoomed-in areas of pericentromeric heterochromatin. (C) Venn diagram of genomic regions enriched with different ChIP methods.

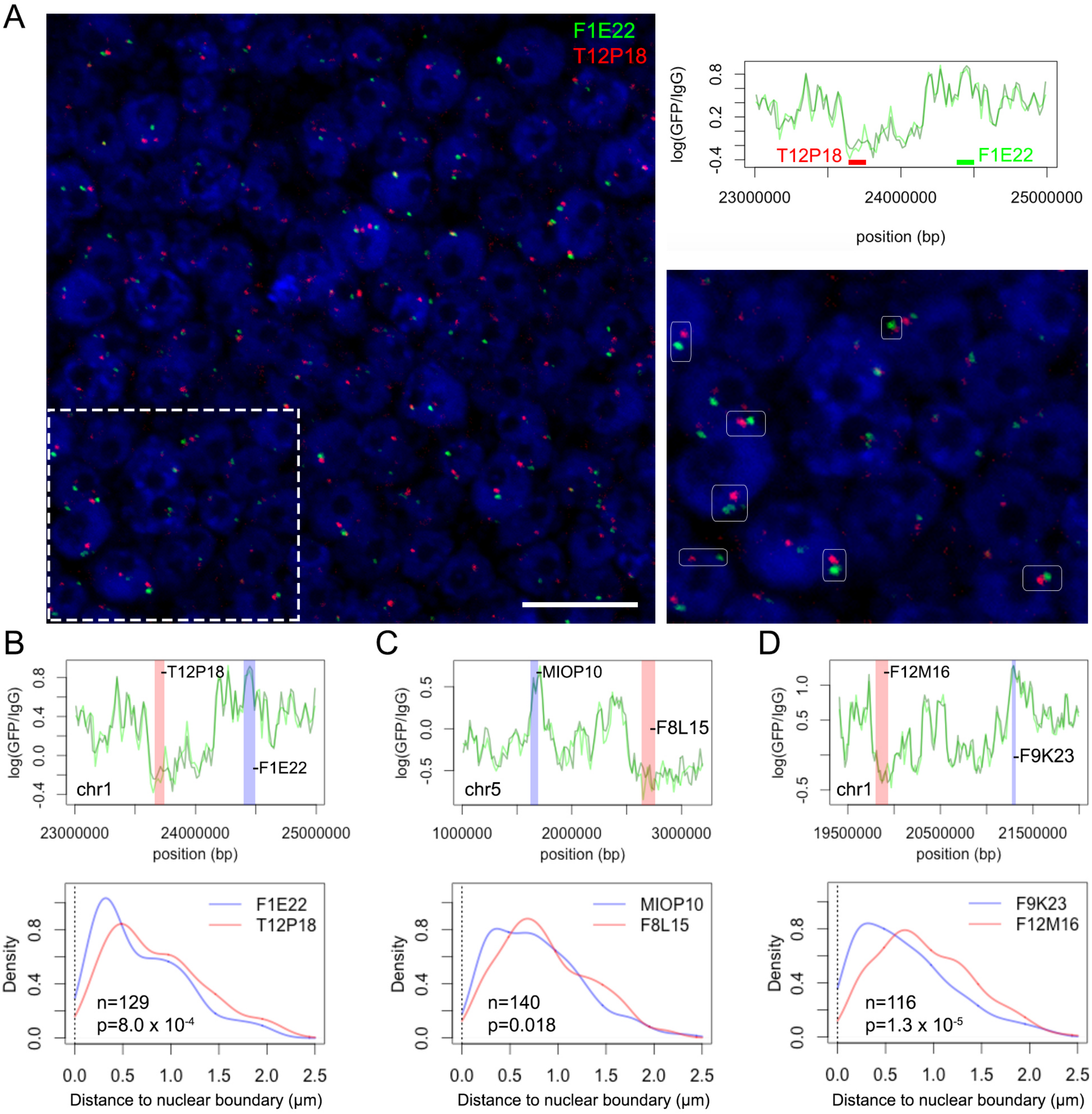

Supplemental Figure S3. DNA FISH.
(A) The plot at the upper right corner shows NUP1:GFP RE-ChIP-seq signal with a duplicate from 7-day-old leaf tissues, and positions of the BAC probes on the genome. The physical distance between BAC probes on the genome is relatively small, such that their signals are often close to each other due to physical linkage. In the zoomed in panel, the outlined areas highlight cases where the green probe is located at the nuclear periphery, whereas the red probe (presumably depicting the control chromatin region on the same chromosome) is located towards the nuclear interior. Scale bar, 10 μm. (B-D) Distribution of the distances between selected chromatin regions with respect to the nuclear boundary. Top panels are the same as in (A). The p-values indicate one-sided Mann-Whitney *U* test results.

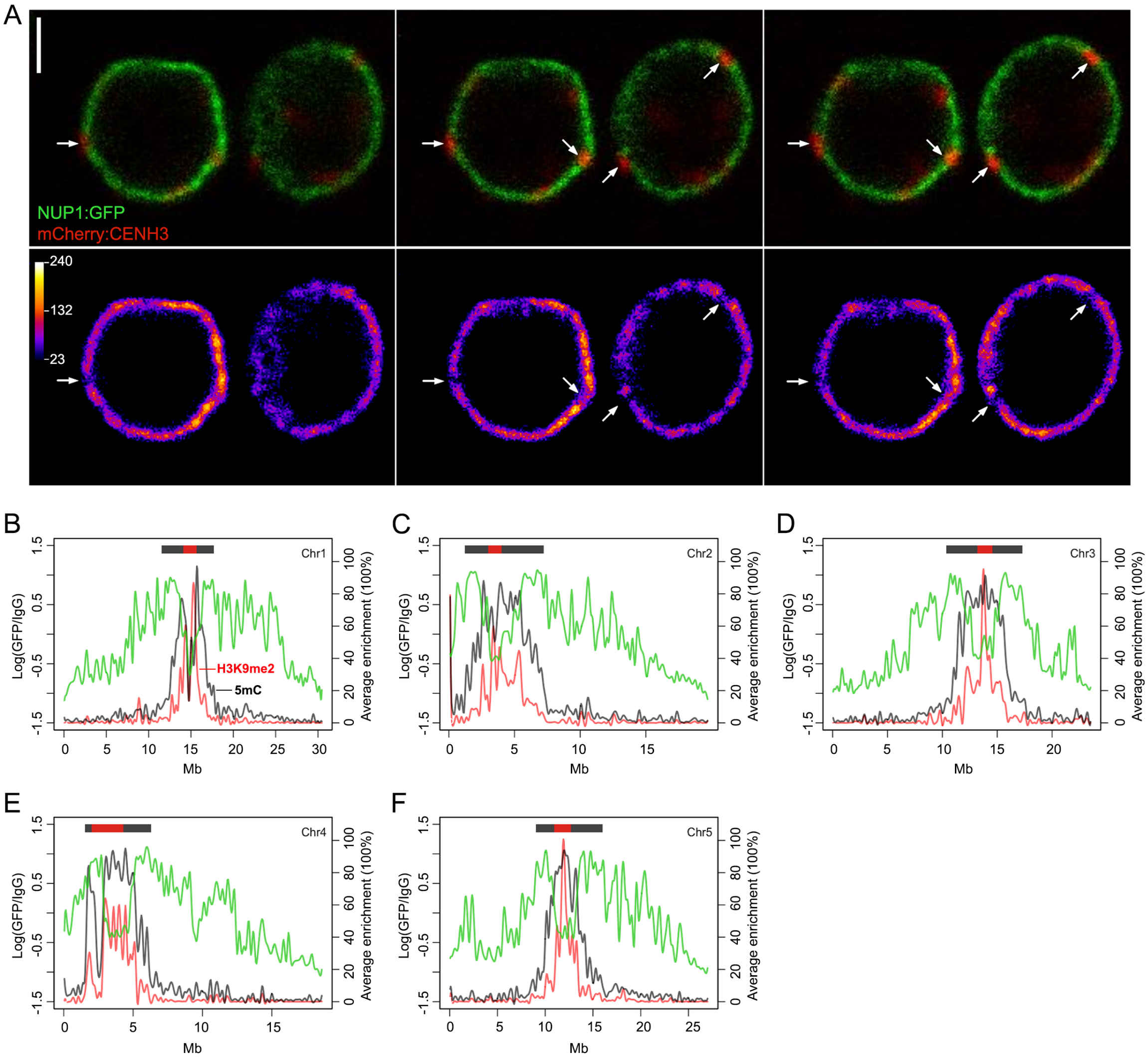

Supplemental Figure S4. NUP1 and CENH3 display complementary patterns at the nuclear periphery.

(A) Three consecutive z-stack confocal images of root cell nuclei showing NUP1:GFP and mCherry:CENH3 patterns. The top row shows NUP1:GFP (green) and mCherry:CENH3 (red) signals. The bottom row shows pixel intensities of the green florescence image. Arrows depict centromeres. Scale bar, 2 µm.

(B to F) Distributions of NUP1:GFP RE-mediated ChIP-seq signals (green line) and heterochromatic marks in five *Arabidopsis thaliana* chromosomes. Plots are according to a 50 kb bin setting. For the H3K9me2 (red line) and DNA methylation (black line) marks, the average enrichment (y-axis on right) means the percentage of regions enriched for the respective epigenetic mark. For all panels, the horizontal bars depict heterochromatic regions, within which centromeric regions are highlighted in red. Epigenetic data is from an integrated *Arabidopsis* epigenome (Wang et al. 2015).

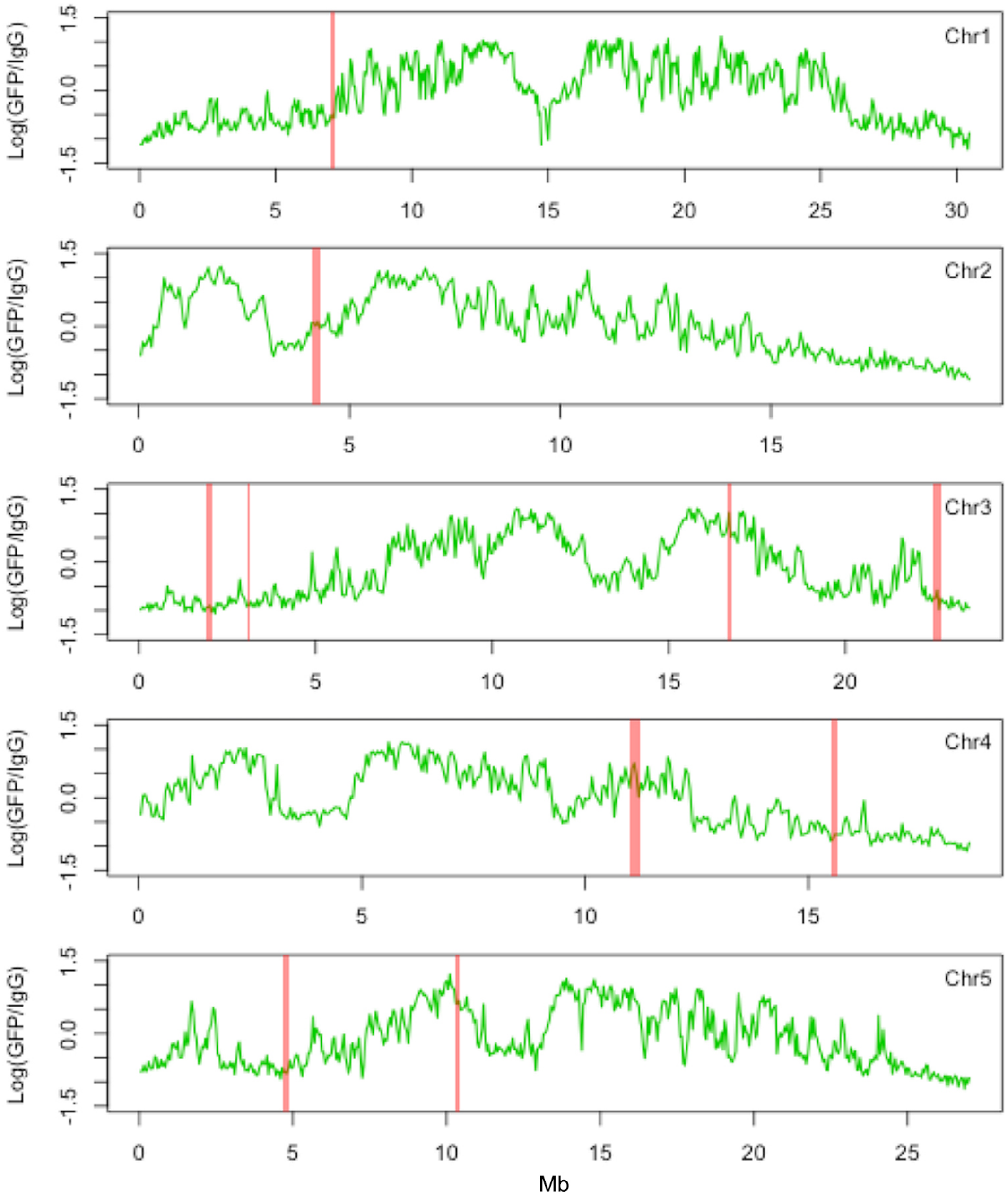

Supplemental Figure S5. NUP1 RE-ChIP-seq signals and IHIs/KEEs.
The red vertical strokes indicate IHIs/KEEs regions (Feng et al. 2014; Grob et al. 2014). Green lines depict NUP1:GFP RE-ChIP-seq signal (50 kb window size), represented as the log2 value of the ratio between normalized anti-GFP and IgG coverage.

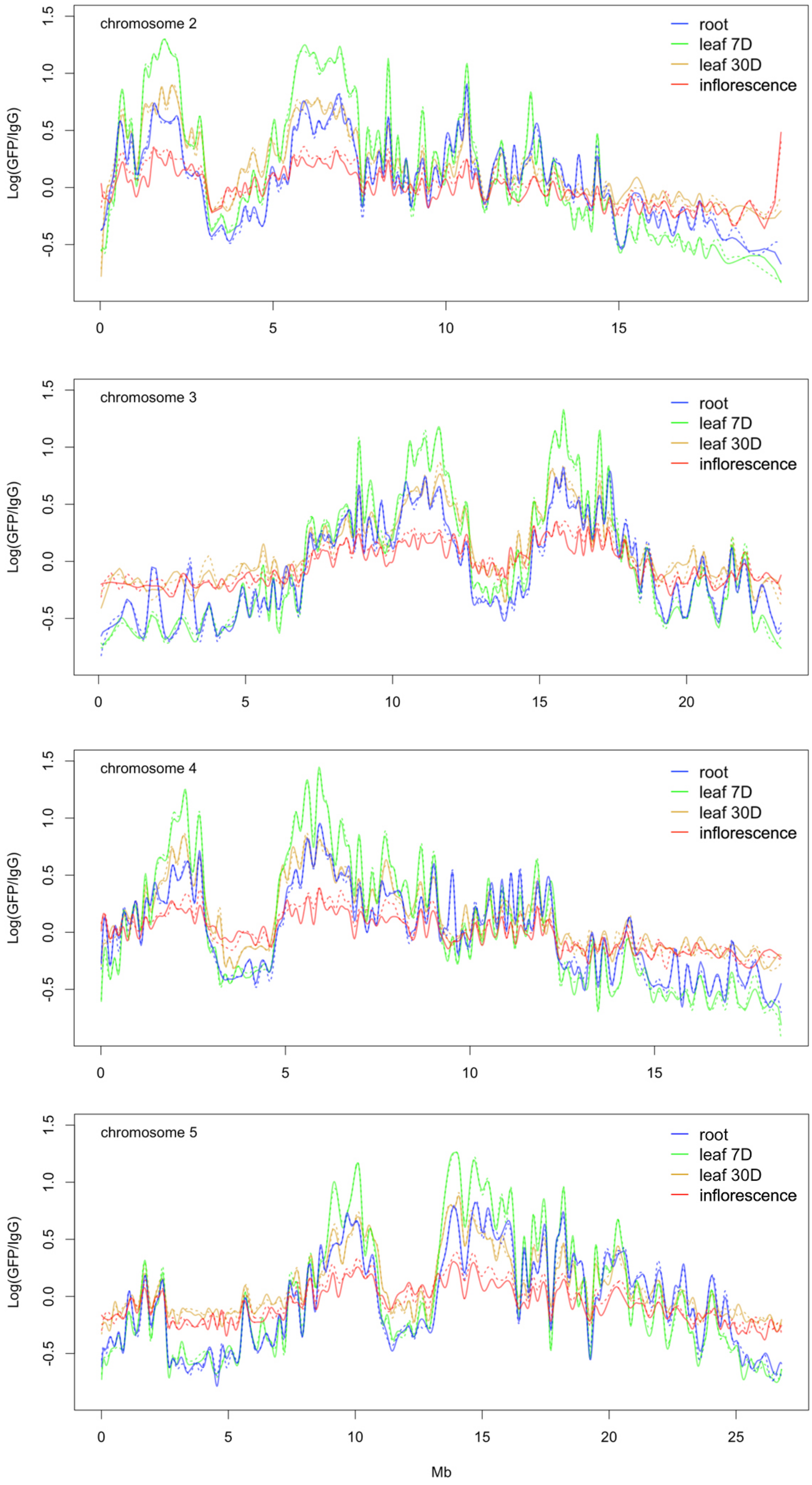

Supplemental Figure S6. NUP1 RE-ChIP-seq signals in different tissues.
Signal of NUP1:GFP RE-ChIP-seq along chromosomes. Labels are the same as in Figure 3A.

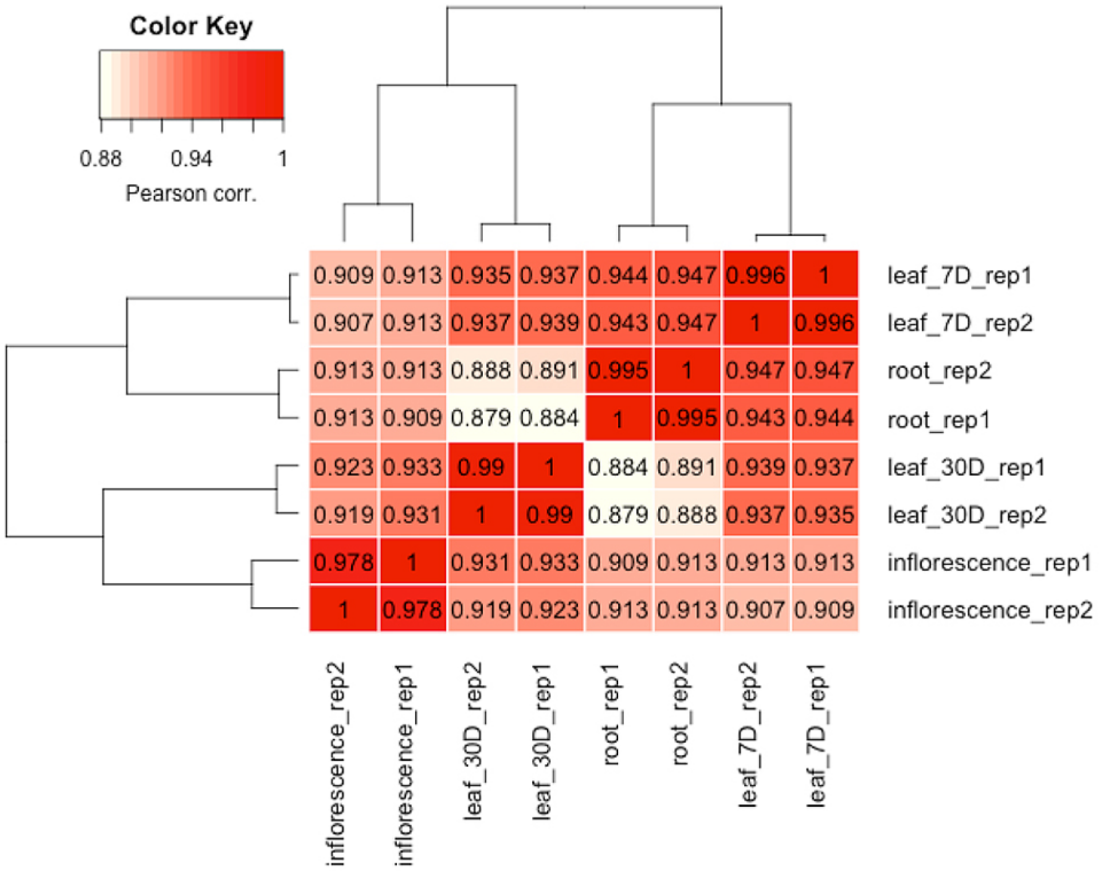

Supplemental Figure S7. Clustering analysis of NUP1 RE-ChIP-seq signals from different tissues.
Clustering of samples is based on normalized sequencing depth (20 kb window size, with centromeric regions omitted). Numbers mean Pearson correlation coefficient.

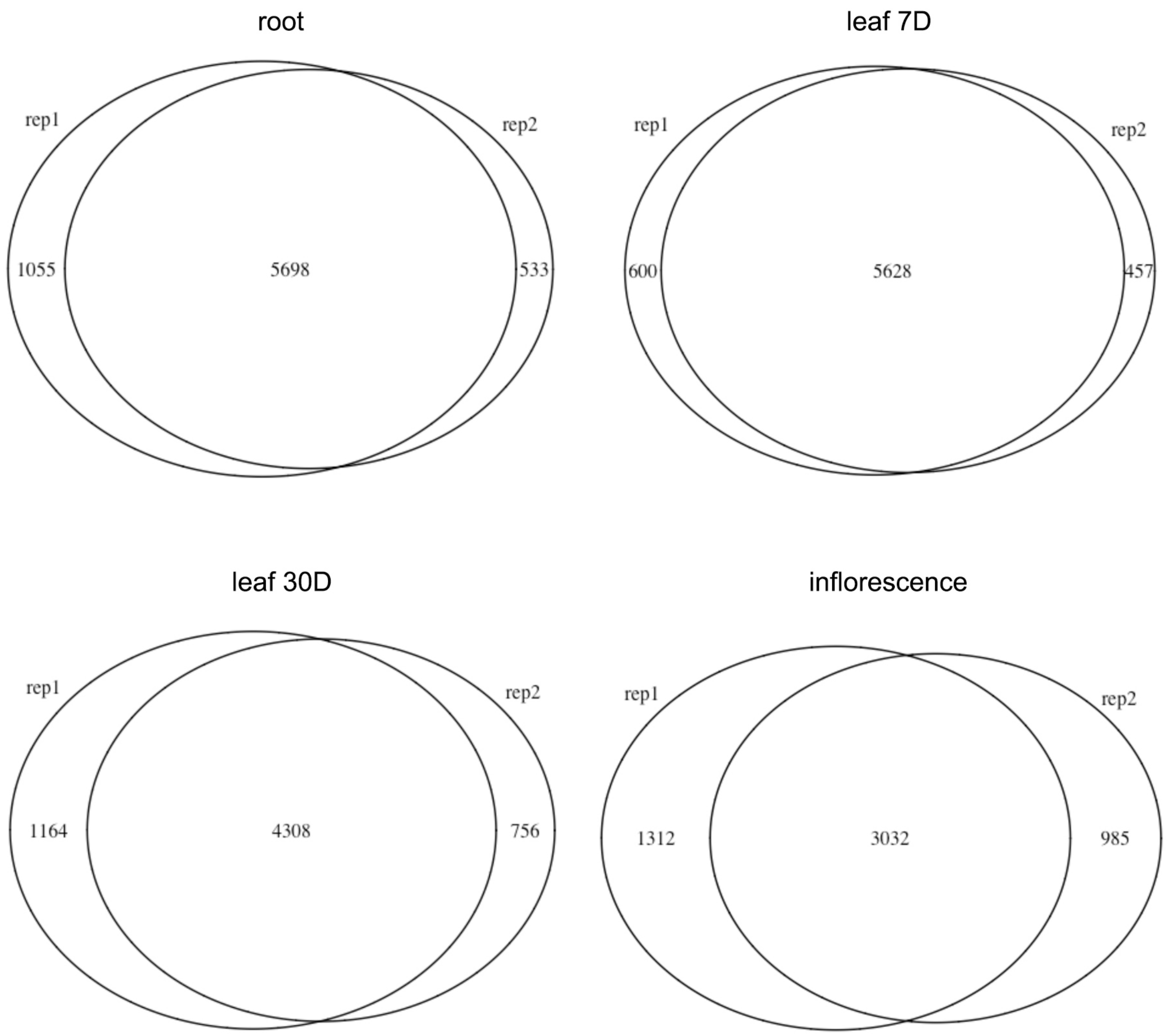

Supplemental Figure S8. Venn diagram of enriched genes.
For each tissue, the number in middle means shared genes in two replicates.

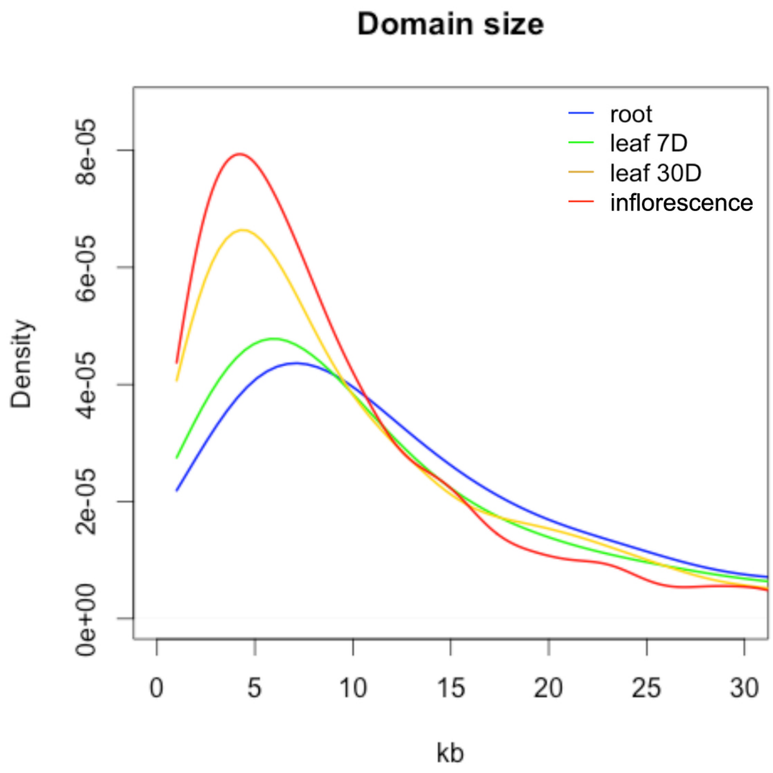

Supplemental Figure S9. Size distribution of NUP1-enriched domains.
Plots are based on the size distribution of shared NUP1-enriched regions in the two replicates.

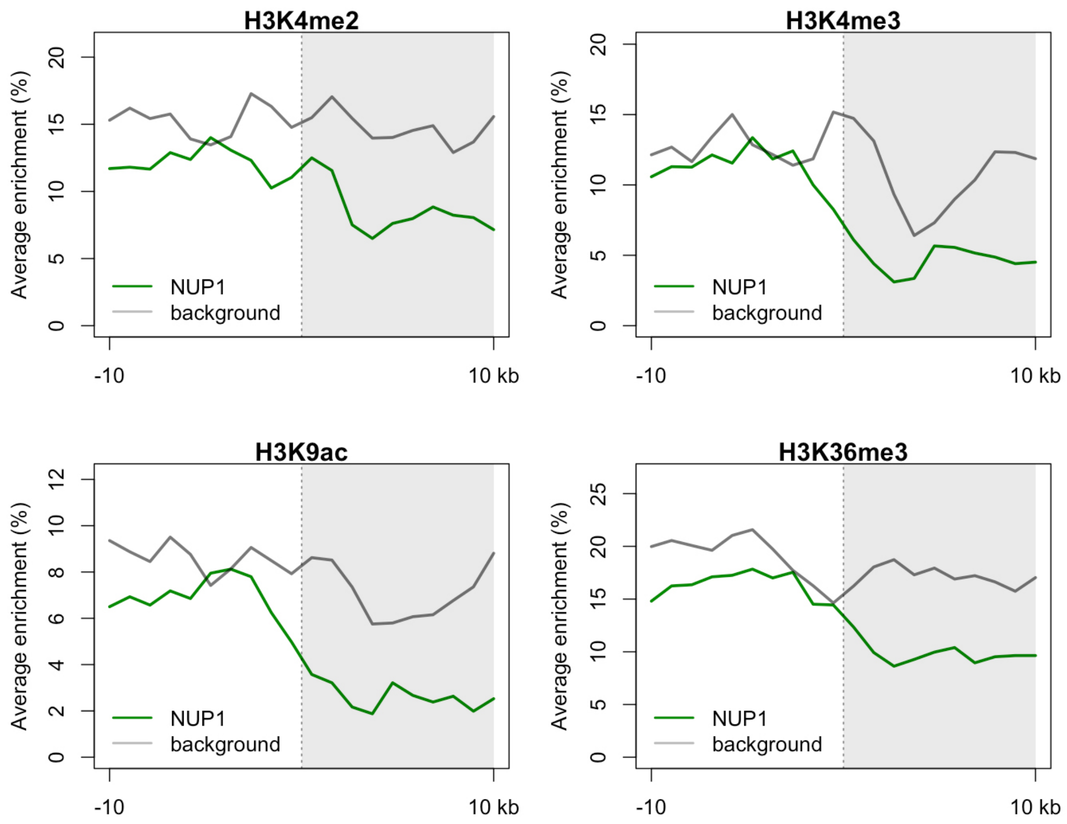

Supplemental Figure S10. Epigenetic marks around NUP1-enriched domain borders. Labels are the same as in Figure 4B.

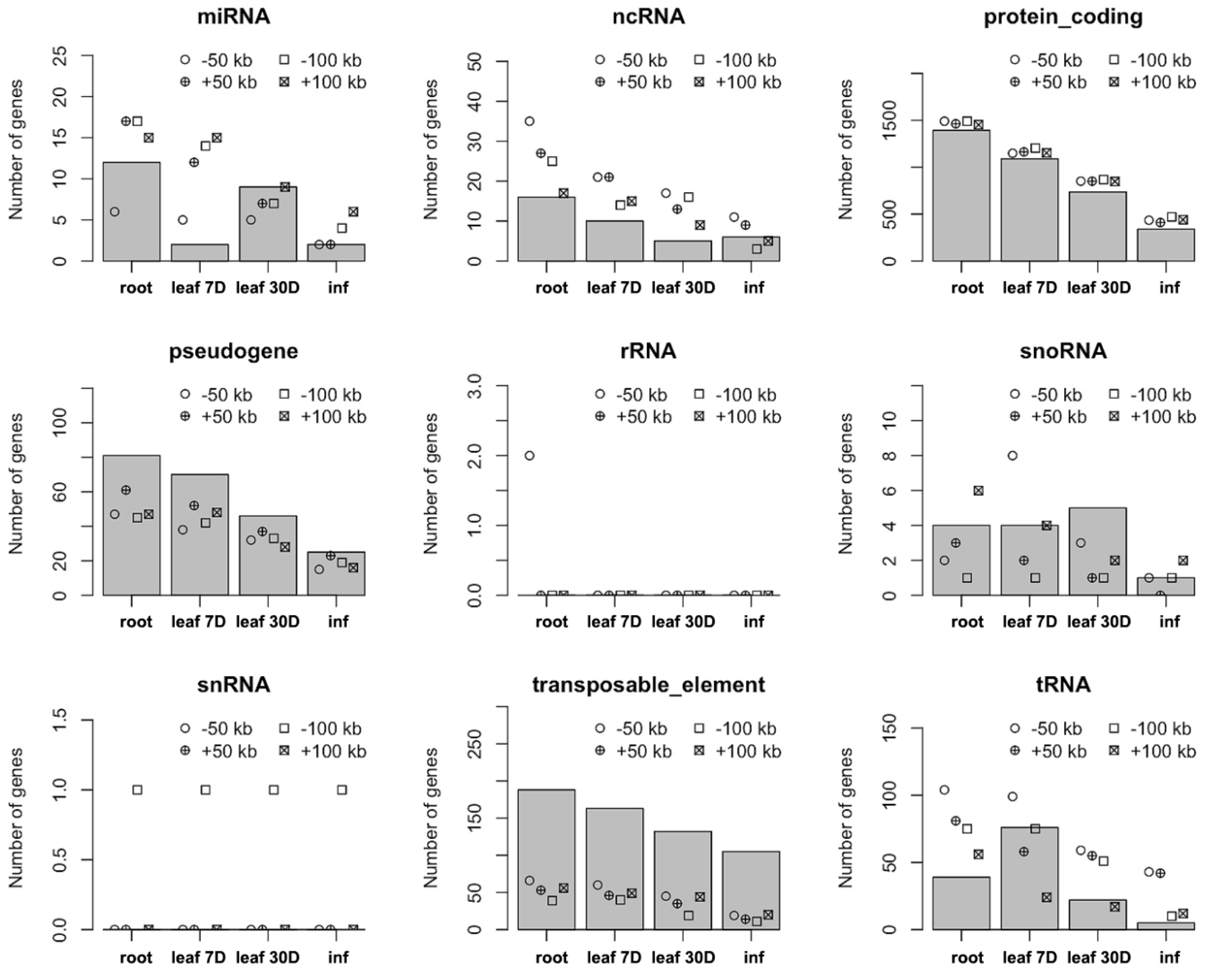

Supplemental Figure S11. Analysis of gene types enriched at nuclear periphery.
For each panel, the labels are the same as in Figure 5A. The two panels corresponding to protein-coding genes and TE genes are shown in Figure 5A.

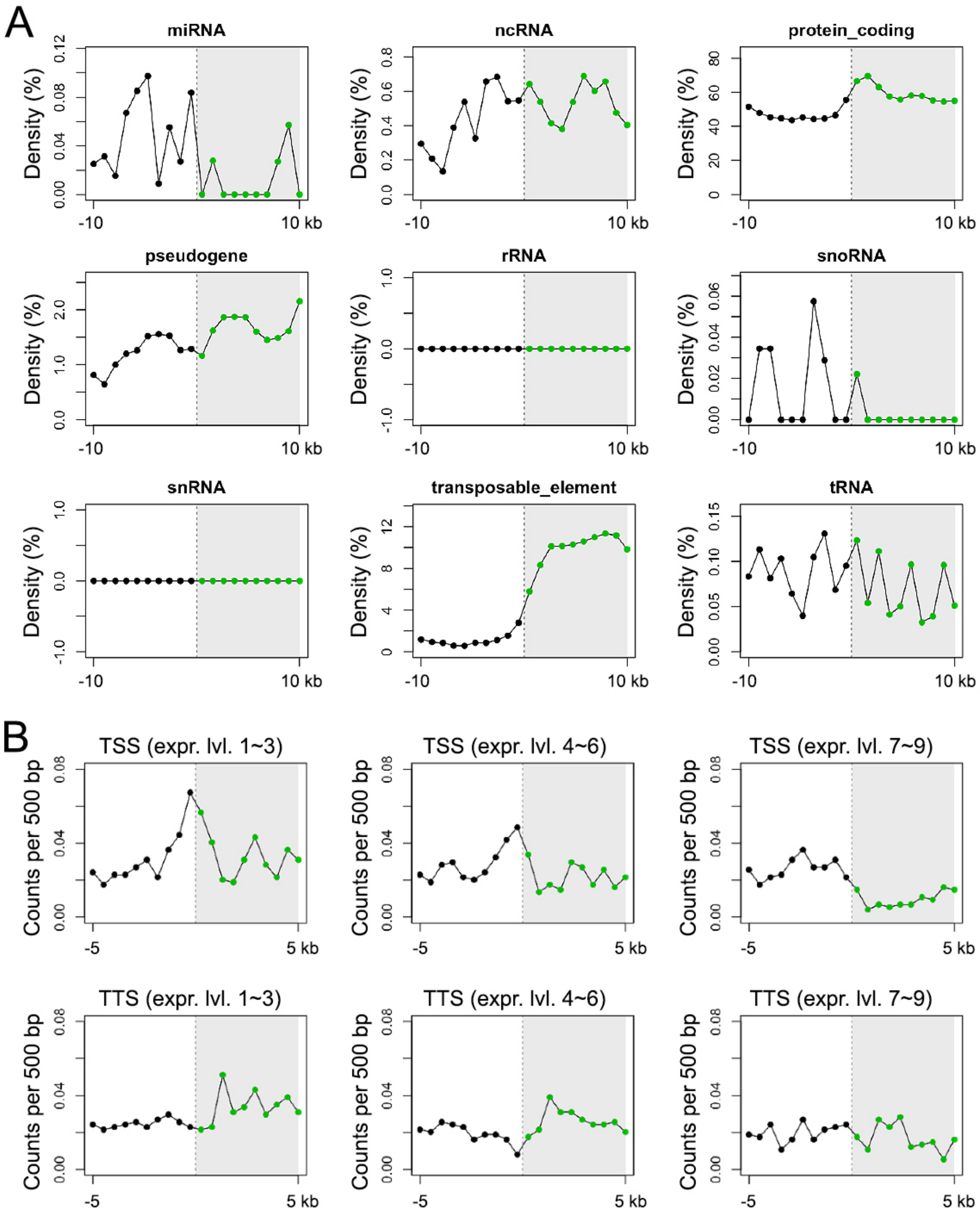

Supplemental Figure S12. Analyses of genomic features around NUP1-enriched domain boundaries.

(A) Distribution of various types of genes around NUP1-enriched domain boundaries. Density means the average percentage of each 1 kb bin annotated with the respective sequence feature. The rest labels are the same as in Figure 4B. (B) Distribution of TSSs (Transcription Start Sites) and TTSs (Transcription Termination Sites), measured as the average number of TSSs or TTSs in a 500 bp bin. Genes were grouped according to their expression levels, where level 1 means the lowest (Wang et al. 2015). The rest labels are the same as in (A).

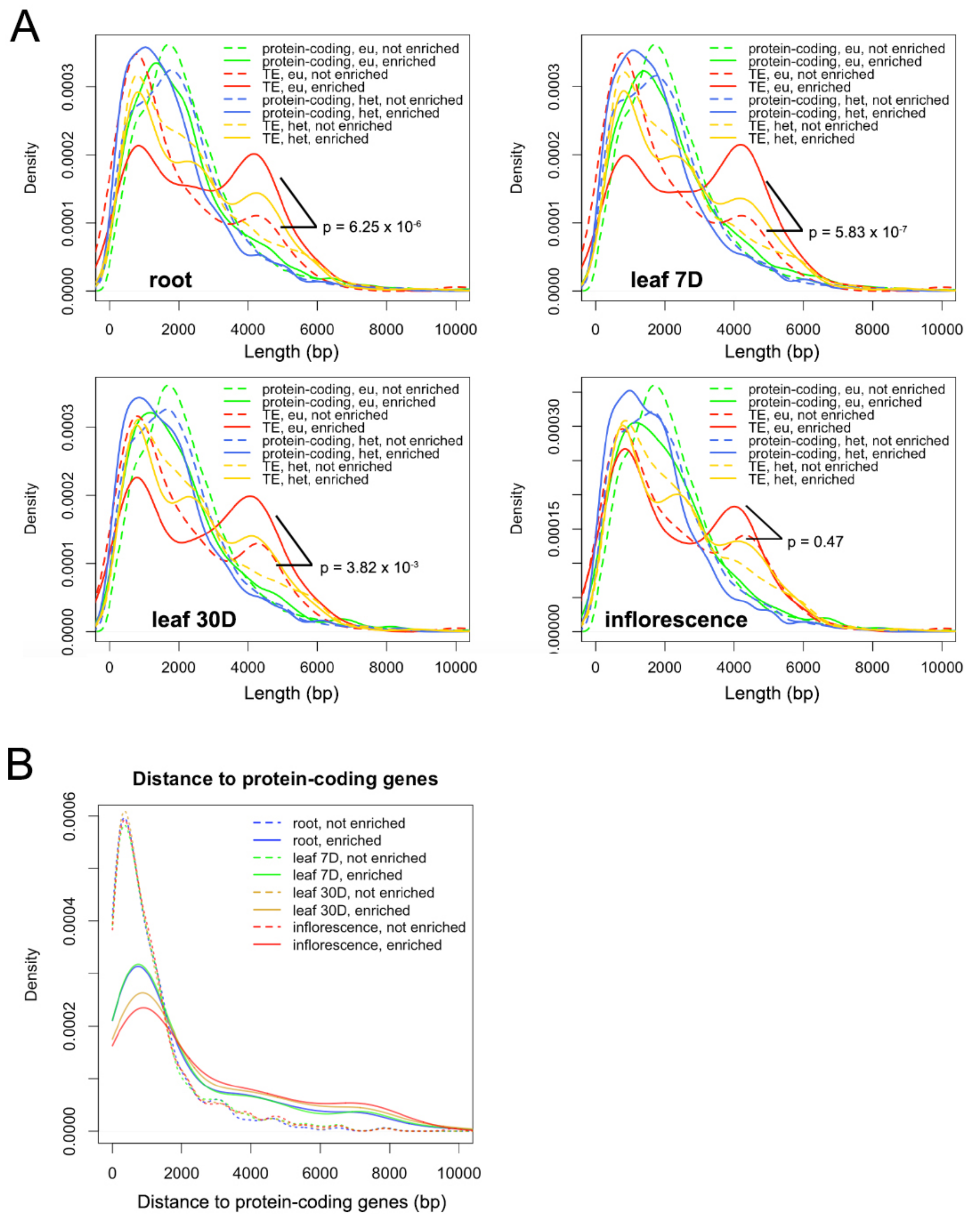

Supplemental Figure S13. TE genes are selectively anchored to the nuclear periphery. (A) Distribution of protein-coding and TE gene lengths. Members of each gene type are grouped according to their location (eu, located on euchromatic arms, which is at least 1 MB from pericentromeric heterochromatin; het, located inside pericentromeric heterochromatin) The p-values indicate Mann-Whitney *U* test results between the red lines. (B) The distance of each TE gene to protein-coding genes is measured as its distance to the nearest protein-coding gene regardless of relative transcription direction. For all tissues, the differences between “enriched” and “not enriched” are significant ($p < 1.0 \times 10^{-6}$ from Mann-Whitney *U* tests).

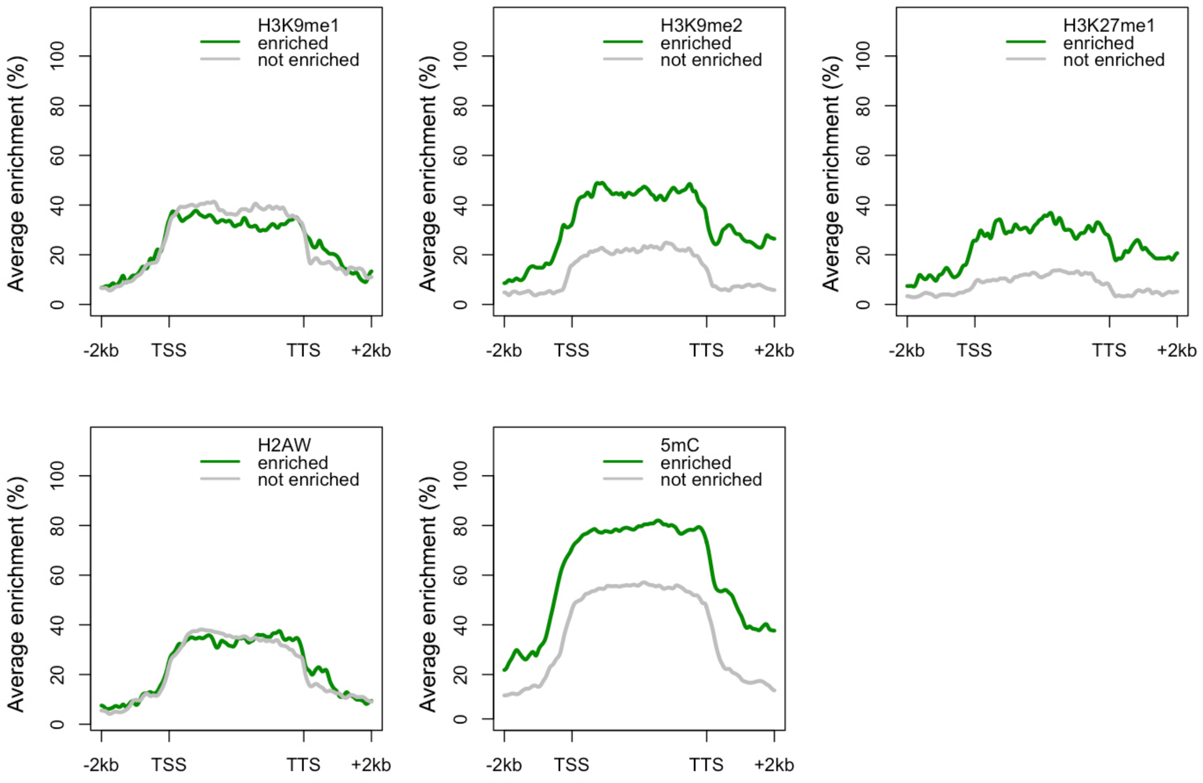

Supplemental Figure S14. Comparison of epigenetic marks around TE genes.
Green and gray lines depict TE genes enriched and not enriched by NUP1, respectively. In each panel, TE genes are linearly scaled to align their TSSs and TTSs. Average enrichment means the percentage of regions (calculated from 100 bp windows) enriched for the respective epigenetic mark.

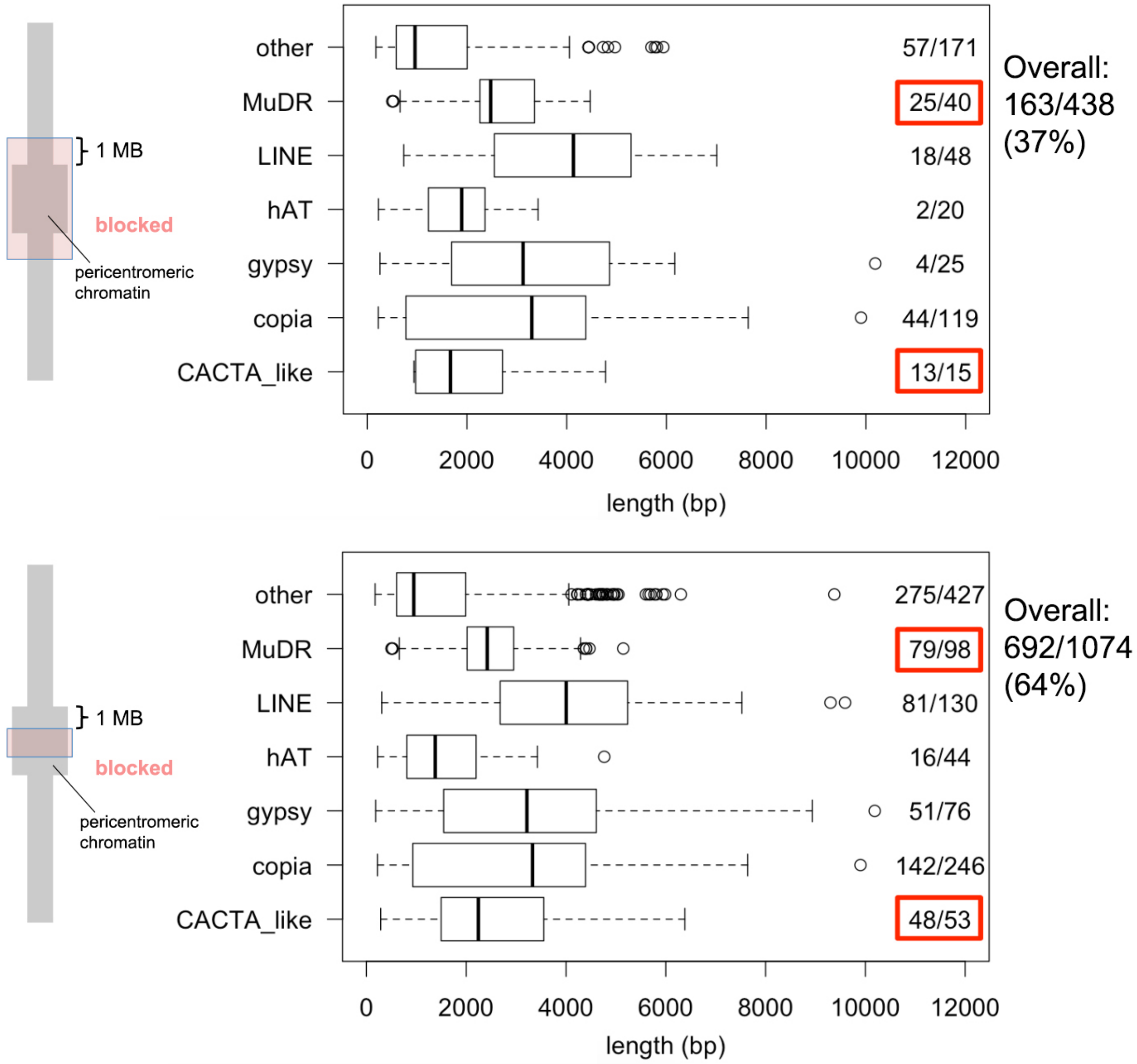

Supplemental Figure S15. Enrichment of different TE gene families at the nuclear periphery.
For each panel, TE genes located in the shaded box shown on left (“blocked”) are not included. The numerators and denominators in the box plots mean numbers of enriched and total numbers of TE genes, respectively. The two outlined TE gene families with top enrichment rates are described in the main text.

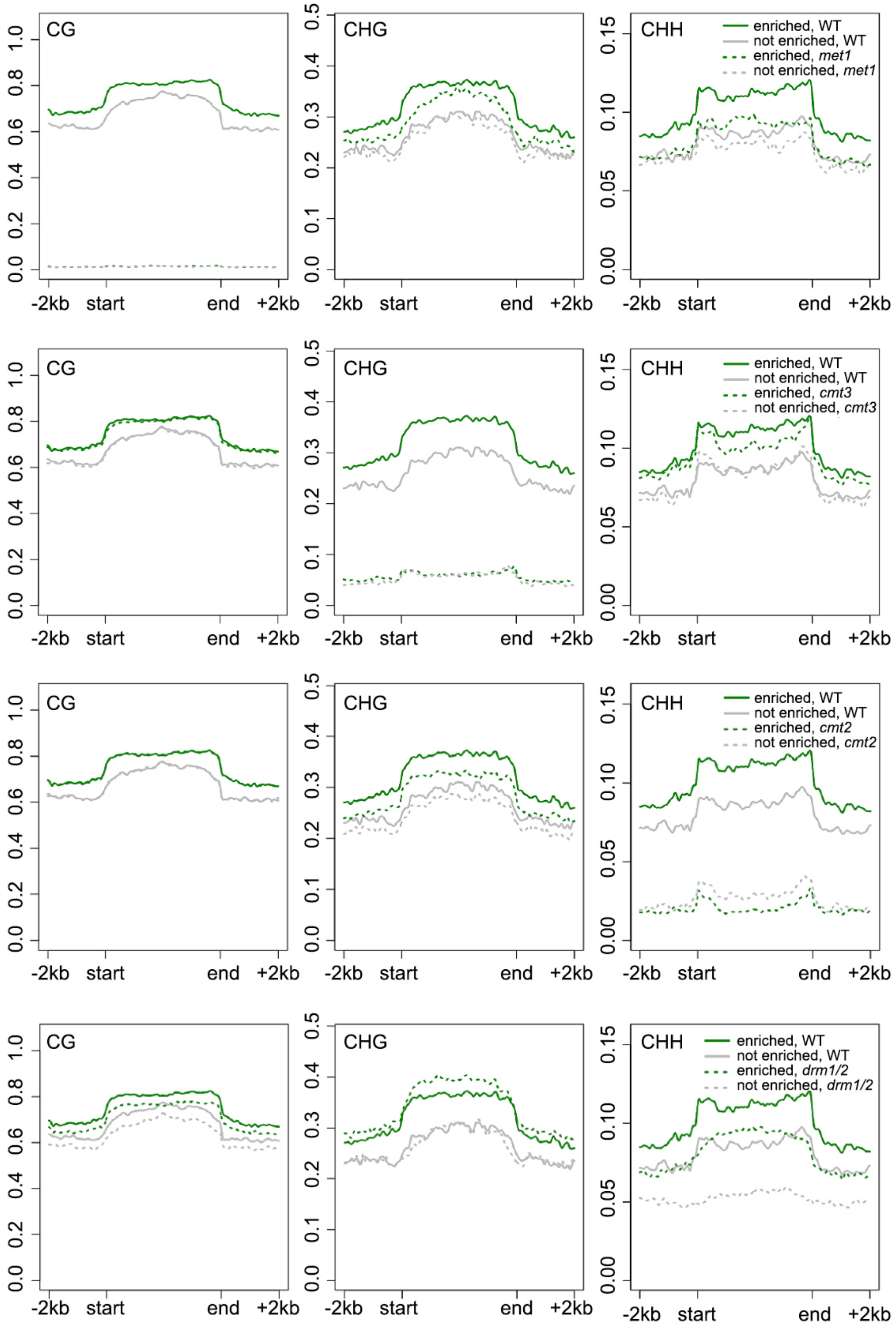

Supplemental Figure S16. Comparison of DNA methylation over TEs.
TEs located inside pericentromeric chromatin are selected. Labels are as in Figure 6.
