# Supplementary material for: Nonrandom domain organization of the Arabidopsis genome at the nuclear periphery
Source: Genome Res. 2017 Jul;27(7):1162–73. doi: 10.1101/gr.215186.116 (PMC5495068; doi:10.1101/gr.215186.116)
Supplement: Supplemental Material [file supp_gr.215186.116_Supplemental_Table_S1.docx]

| Sample | Raw reads | Mapped reads |
| --- | --- | --- |
| Regular ChIP | | |
| leaf_7D_GFP | 29,112,043 | 26,269,989 |
| leaf_7D_IgG | 22,505,801 | 19,903,273 |
|  |  |  |
| RE-mediated ChIP (trial1) | | |
| leaf_7D_GFP_mild | 4,356,381 | 3,774,643 |
| leaf_7D_IgG_mild | 4,051,577 | 3,534,263 |
| leaf_7D_GFP_very_mild | 4,150,919 | 3,585,992 |
| leaf_7D_IgG_very_mild | 4,243,746 | 3,591,349 |
|  |  |  |
| RE-mediated ChIP (trial2) | | |
| Col-0_H3K9me2_rep1 | 11,311,406 | 10,468,807 |
| Col-0_H3K9me2_rep2 | 13,895,221 | 12,985,863 |
| Col-0_IgG_rep1 | 8,619,667 | 7,386,643 |
| Col-0_IgG_rep2 | 9,085,510 | 7,679,964 |
|  |  |  |
| RE-mediated ChIP | | |
| root_GFP_rep1 | 18,271,323 | 14,635,135 |
| root_GFP_rep2 | 14,788,390 | 10,736,901 |
| root_IgG_rep1 | 13,182,145 | 11,146,699 |
| root_IgG_rep2 | 14,724,600 | 11,723,771 |
| leaf_7D_GFP_rep1 | 15,330,888 | 12,859,124 |
| leaf_7D_GFP_rep2 | 17,395,095 | 15,648,196 |
| leaf_7D_IgG_rep1 | 16,492,325 | 14,335,355 |
| leaf_7D_IgG_rep2 | 15,633,267 | 13,063,288 |
| leaf_30D_GFP_rep1 | 15,191,514 | 13,694,876 |
| leaf_30D_GFP_rep2 | 11,597,466 | 9,670,522 |
| leaf_30D_IgG_rep1 | 10,866,188 | 9,517,570 |
| leaf_30D_IgG_rep2 | 10,817,251 | 9,048,152 |
| inflorescence_GFP_rep1 | 16,815,632 | 14,518,475 |
| inflorescence_GFP_rep2 | 19,482,233 | 17,890,959 |
| inflorescence_IgG_rep1 | 12,663,466 | 10,962,140 |
| inflorescence_IgG_rep2 | 11,605,240 | 9,991,679 |

Supplemental Table S1. Statistics of sequencing reads.
