# Supplementary material for: Nonrandom domain organization of the Arabidopsis genome at the nuclear periphery
Source: Genome Res. 2017 Jul;27(7):1162–73. doi: 10.1101/gr.215186.116 (PMC5495068; doi:10.1101/gr.215186.116)
Supplement: Supplemental Material [file supp_gr.215186.116_Supplemental_Table_S4.docx]

| Product name | Sequences (5’ --> 3’) |
| --- | --- |
| TUB2 | AAGAACCATGCACTCATCAGC |
|  | ATCCGTGAAGAGTACCCAGAT |
| AT3G10114 | CTTAAGAAGGGAGAGCTGAT |
|  | CACTTTGTCTCACGAAGCTC |
| AT2G24890 | TAGGGAAATCGAAAGAATCG |
|  | AACTCAGTCTTAAGCCTTTC |

Supplemental Table S4. Primers used for ChIP-qPCR
